# A Descriptive Primary Cell-Based In Vitro System to Model Interactions Between *Lactobacillus* Extracellular Vesicles and Colonic Epithelium

**DOI:** 10.3390/microorganisms14102367

**Published:** 2026-10-15

**Authors:** Deepalakshmi Govindarajan, Ulrike Rolle-Kampczyk, Nico Jehmlich, Martin von Bergen, Steven Schulte, Karl-Herbert Schäfer, Christian Bökel, Christian Tontsch, Barbara Spellerberg, Thomas Seufferlein, Viktoria Hentschel

**Affiliations:** 1 Department of Internal Medicine 1, University Hospital Ulm, 89081 Ulm, Germany; deepalakshmigovindarajan@gmail.com (D.G.);; 2 Department of Molecular Toxicology, Helmholtz Centre for Environmental Research, 04318 Leipzig, Germany; 3 Institute of Biochemistry, University Leipzig, 04103 Leipzig, Germany; 4 German Center for Integrative Biodiversity Research Leipzig, 04103 Leipzig, Germany; 5 Informatics and Microsystems Technology, University of Applied Sciences Kaiserslautern, 66482 Zweibrücken, Germany; 6 Core Facility for Confocal and Multiphoton Microscopy, Ulm University, 89081 Ulm, Germany; 7 Institute for Organic Chemistry 1, Ulm University, 89081 Ulm, Germany; 8 Institute of Medical Microbiology and Hygiene, Ulm University, 89081 Ulm, Germany

**Keywords:** extracellular vesicles, lactic acid bacteria, lipoteichoic acid, colonic organoids, proteomic mass spectrometry

## Abstract

Bacterial extracellular vesicles (EVs) have emerged as a relevant mode of communication between host and resident microbiota. Evidence is limited regarding the ability of Gram-positive EVs to interact with primary colonic epithelial cells. We propose a murine organoid-based in vitro model to study the impact of EVs from the lactic acid bacteria species *Limosilactobacillus reuteri* (LR) and *Lactiplantibacillus plantarum* (LP) on primary colonic epithelium. Previous EV characterization revealed significant differences in zeta-potential (LR: −0.0185 V vs. LP: −0.0103 V, *p* 0.0125) and particle concentration (LR: 3.38 × 10^8^ particles/mL vs. LP: 8.32 × 10^8^ particles/mL, *p* 0.0029) but not in lipoteichoic acid (LTA) content and long-chain fatty acid composition. Applied to colonic organoids, uptake efficiency differed between LR and LP EVs, with LR exhibiting higher internalization rates than LP (5 µg EV, 46.56% vs. 20.03%, *p* < 0.0001) at the expense of cellular viability. Internalized EVs partially co-localized with endo-lysosomal compartments and, depending on the bacterial species of origin, induced a proteomic stress response mimicking molecular features of neurodegenerative diseases. Proteomic profiling of EVs and parental bacteria revealed the existence of lipoproteins predicted to be embedded in the cytoplasmic membrane, potentially acting as adhesion factors to the epithelium. Pre-emptive blocking with LTA reduced EV uptake into the epithelium (1 µg/mL LTA: 3.77 × 10^5^ A.U. vs. control: 1.02 × 10^6^ A.U., *p* 0.0231). Efficiency of EV internalization differs between LR and LP and may be associated with LTA decoration. Studies using LTA-mutant *Lactobacillus* species are warranted to clarify the role of vesicle-bound LTA in the EV uptake machinery.

## 1. Introduction

Bacteria-derived extracellular vesicles (EVs) are increasingly recognized as key mediators of communication, not only among members of the microbiota but also between bacteria and host cells. These nanoscale vesicles are encapsulated by a lipid bilayer and carry a complex cargo mainly composed of proteins and nucleic acids [1]. The ability to produce and secrete EVs is considered a widespread phenomenon across both commensal and pathogenic bacterial species [2]. In addition to mediating inter-microbiota interactions essential for processes such as quorum sensing, biofilm formation, nutrient acquisition, and bactericidal functions, bacterial EVs can exert diverse and sometimes opposing effects on the host, depending on their microbial source [3,4,5].

To date, research covering such inter-kingdom communication specifically between gut bacterial EVs and mammalian host cells has focused on colonic epithelial cell models, most of which incorporate immortalized colorectal cancer cell lines to represent the epithelial lining [6,7,8]. Applied to the well-characterized human Caco-2 cell line, EVs from the Gram-positive and -negative species *Lacticaseibacillus casei*, *Enterococcus faecalis*, and *Proteus mirabilis* induce differential expression of the chemokines *CCL20*, *CXCL8*, and *CXCL10* and microbial sensing receptors NOD2, NLRP1, TLR3, or TLR7 mediated by their RNA cargo [9] while EVs from *Escherichia coli* strain Nissle 1917 inhibit expression of the oligopeptide transporter PepT1 via upregulation of miR-193a-3p [7].

However, given the aberrant genetic and functional traits of these transformed lines, their reliability in modeling the native epithelial response remains uncertain. Organoids, which recapitulate the architecture and function of the intestinal epithelium in vivo, offer a physiologically relevant alternative for studying EV uptake and intracellular trafficking. Due to foreseeably restricted access to human colonic biopsies, colonic organoids derived from adult C57BL/6J mice were utilized in lieu of human-derived organoids, enabling a reproducible and scalable experimental system to unveil general mechanisms of the mammalian host epithelial response to bacterial EVs.

Numerous lactic acid bacteria (LAB) members are nowadays exploited by food industries for their probiotic benefits, which include effective colonization of the gastrointestinal tract and repulsion of pathogenic species, bacteriocidal activities, and antioxidant and immunomodulatory effects [10,11,12]. A constantly growing number of reports document the ability of several *Lactobacillus* species to produce EVs [13,14,15,16,17]. To date, EVs derived from various sources, including *Limosilactobacillus reuteri* DSM 17938 and BG-R46, and *Lactiplantibacillus plantarum* HY7714, have primarily been implicated in the stabilization of cell–cell junctions and the activation of immune-related intracellular signaling pathways in in vitro studies of intestinal epithelial cells [13,18,19]. However, a recurrent feature of these studies is that EVs from a single species were investigated.

To comparatively study the interaction between bacterial EVs and colonic epithelium, we opted for *Limosilactobacillus reuteri* DSM 20016 and *Lactiplantibacillus plantarum* subspecies *plantarum* DSM 20174, belonging to different genera and species but stemming from the same family of *Lactobacillaceae*. These species were chosen as study models to represent the resident gut microbiota and food-borne environmental sources, respectively. *Limosilactobacillus reuteri* strain DSM 20016 is the type strain of the ancient *Lactobacillus fermentum* subspecies *reuteri* previously isolated from the human intestine. Besides its cobalamin-synthesizing capacities, it is particularly known for its ability to produce reuterin, showing microbicidal effectiveness against several bacterial and fungal microorganisms [20,21,22]. *Lactiplantibacillus plantarum* subspecies *plantarum* strain DSM 20174 was cultivated from pickled cabbage and, upon ingestion by mice, was shown to confer protection against progression of non-alcoholic fatty liver disease by reducing white adipose tissue inflammation and altering macrophage polarization [23,24].

In this study, we investigated how primary colonic epithelial cells devoid of surrounding immune and stromal cells respond to bacterial EVs at varying dose levels, with a particular focus on proteomic profiling. We utilized two different yet taxonomically related Gram-positive bacterial species to compare EV adhesion to and uptake into the host cell. Additionally, EVs were extensively characterized to identify features that may affect surface properties and might explain species-specific predilections of EVs towards the host epithelium.

With this primary cell-based in vitro experimental system, we aim to further characterize the as-yet ill-defined interactions of Gram-positive bacterial EVs with colonic epithelium by addressing major events including EV binding and uptake, intracellular distribution, and biological impact on host cells.

## 2. Materials and Methods

### 2.1. Bacterial Culture and EV Isolation

*Limosilactobacillus reuteri* (DSM 20016) (LR) and *Lactiplantibacillus plantarum* subsp. *plantarum* (DSM 20174) (LP) was purchased from Deutsche Sammlung für Mikroorganismen und Zellkulturen (DSMZ), Braunschweig, Germany. Bacteria were grown in De Man-Rogosa-Sharpe (MRS) broth (69966, Merck-Millipore, Burlington, MA, USA) supplemented with Tween 80 (P1754, Sigma-Aldrich, St. Louis, MO, USA) at 37 °C. An overnight starter culture was used to inoculate 50 mL and 500 mL of pre-warmed MRS broth for small-scale and large-scale EV isolation, respectively. Bacterial culture was terminated within an optical density (OD)_600_ range of 4.0–5.0 before reaching the stationary growth phase. The culture supernatant was separated by three consecutive centrifugation steps (300× *g*, 4 °C, 10 min), followed by clearing off residual bacterial cells at a higher centrifugation speed (7000× *g*, 4 °C, 30 min). The supernatant was sterile-filtered through a syringe filter (83.1826.001, Filtropur S, Sarstedt, Nümbrecht, Germany) or a vacuum-driven bottle top filter (430771, Corning, NY, USA) with a 0.2 µm pore size. EVs were isolated portion-wise from the supernatant by ultracentrifugation with an Optima XL-100K (Beckman-Coulter, Brea, CA, USA) equipped with a fixed-angle Ti60 rotor (k-factor 45), for 1 h at 130,000× *g*, 4 °C. The resulting EV pellets were dissolved in DPBS and pooled together for final ultracentrifugation with an Optima MAX-XP (Beckman Coulter, Brea, CA, USA), using a fixed-angle MLA-130 S/N 18U1547 rotor (k-factor 8.7). EVs were solubilized in 100 µL DPBS, corresponding to a 1:10,000 concentration factor, and stored at −80 °C until further use. Contamination with residual living bacteria was excluded by inoculating MRS agar plates (11723553, BD Difco, Franklin Lakes, NJ, USA) with EV suspension of either species. EV biomass protein concentration was determined by colorimetric Bradford assay (5000006, Bio-Rad, Hercules, CA, USA). For all downstream cell culture experiments, concentrated EV preparations referred to as EVs were resuspended in fetal bovine serum (FBS)-free recombinant growth factor medium to avoid FBS as a source of contaminating vesicles.

### 2.2. Cultivation of Murine Colonic Organoids and Organoid-Derived Monolayers

#### 2.2.1. Organoids

Eight- to 12-week-old C57BL/6j male mice bred by the animal research center of Ulm University were used to grow colonic organoids. Animals were housed in specific pathogen-free cages in a temperature- and humidity-controlled environment (25 °C, 40–50% humidity) with a 10/14 h light/dark cycle and ad libitum access to water and standard chow. All animal experiments were approved by the regional county government office (Tübingen, Baden-Württemberg, Germany, license no. o.197-5.TschB:W) and the care for the animals was provided in accordance with institutional guidelines. For production of Wnt3a-, Noggin, and R-Spondin 3-conditioned medium (WNR), stable-transfected L-WNR cells were purchased from AddexBio, San Diego, CA, USA (S0011002).

Following CO_2_ euthanasia, the colon was mobilized from the abdominal cavity, longitudinally opened, and washed with DPBS. The colon was minced with scalpels and cleaned with cold DPBS in a conical tube by inverting it several times and allowing the tissue fragments to settle for 1 min. Following aspiration of the supernatant, this step was repeated up to 5 times until the supernatant had turned clear. Crypts were liberated by enzymatic digestion with collagenase type I (17100-017, Gibco, Waltham, MA, USA) with some modifications to a previously published method article [25]. Following a 30 min period of digestion, a 1000 µL micropipette tip was mounted on a 10 mL serological pipette and coated with 1% FBS/DPBS. Tissue fragments were pipetted up and down 20 times for bulk release of intact crypts. After adding 5 mL of washing medium (Advanced DMEM/F12 supplemented with FBS 10%, penicillin 100 U/mL/streptomycin 0.1 mg/mL, HEPES, L-glutamine 2 mM) prepared according to [25], crypts were sequentially filtered through a 100 µm- and a 70 µm cell strainer, and centrifuged for 5 min (400× *g*, 4 °C). Crypts were resuspended in Matrigel Basement Membrane Matrix (354234, Corning, NY, USA), plated in 15 µL-droplets in a 24-well plate, and incubated at 37 °C for 20 min to allow polymerization of Matrigel. The solidified crypt-Matrigel domes were overlaid with 500 µL WNR-conditioned medium supplemented with 10 µM Y-27632 (Y0503, Sigma-Aldrich, St. Louis, MO, USA) as previously described [25], and cultivated at 37 °C, 5% CO_2_. Colonic organoids were passaged for the first time after 72 h of culture. Briefly, the medium was aspirated and domes were rinsed with 0.5 mM EDTA/DPBS. Then, 200 µL trypsin-EDTA 0.5% (15400-054, Gibco, Waltham, MA, USA) was added and the domes were lifted off by horizontal scratching. Following incubation at 37 °C for 3 min, 500 µL washing medium was added to each well to stop enzymatic digestion. Resuspended colonic organoids were collected and mechanically disrupted by forceful pipetting. Organoid fragments were centrifuged for 5 min at 400× *g*, 4 °C, and resuspended in Matrigel for passaging.

#### 2.2.2. Organoid-Derived Monolayers

Colonic organoids were released from Matrigel with Cell Recovery Solution (354253, Corning, NY, USA), collected in a coated 15 mL conical tube, counted, and adjusted to a concentration of 250,000 organoids per mL. Following centrifugation, the pellet was mildly digested with 500 µL TrypLE (12604-013, Gibco, Waltham, MA, USA) for 8 min at 37 °C and triturated 20 times to disrupt organoids into single cells. For trypsin inactivation, 500 µL of 10 µM Y-27632/L-WNR medium was added to the single-cell suspension. Previously, 8-well chamber slides (94.6190.802, Sarstedt, Nümbrecht, Germany) were coated with an ultra-thin layer of 2% Matrigel/DPBS to promote planar growth. Two hundred µL of organoid single-cell suspension were added to each well and cultivated overnight at 37 °C, 5% CO_2_. Colonies of epithelial monolayers started developing as early as 3 h from culture onset and were cultivated for at least 48 h before experiments were conducted.

### 2.3. Nanoparticle Tracking Analysis

EV concentrations and size distributions were determined with a NanoSight LM10 system NS300 (Malvern Pananalytical Ltd., Great Malvern, Worcestershire, UK) using NTA 3.4 Nanoparticle Tracking and Analysis software. Prior to analysis, EV samples isolated from 50 mL of culture supernatant were diluted 1:300 in DPBS, acclimatized to ambient temperature and briefly vortexed before loading into a 2 mL syringe. Focus, detection threshold, and gain settings were optimized for data acquisition. Per sample, 60 s video sequences were recorded in triplicate at room temperature (22.8 °C) and analyzed using auto particle detection and preset tracking parameters (blur size, max jump distance).

### 2.4. Zeta-Potential Measurement

Electrophoretic light scattering (ELS) was performed on a Litesizer DLS 700 equipped with a laser light source of 658 nm/40 nW (Anton Paar, Graz, Austria) to measure the zeta-potential (ζ-potential). For data analysis, the software package Kalliope Professional (v3.8.1) was employed. EVs were solubilized in 5% dextrose solution supplemented with 1 mM NaCl and frozen at −80 °C, not exceeding 2 weeks of storage. Fifty µL of EV sample volume was thawed, loaded into the low-volume accessory of the Univette cell, allowed to equilibrate to ambient temperature for 5 min, and heated to 37 °C inside the LiteSizer device. After tempering the Univette containing the sample, a delay of 3 min was set before measurements were launched. A forward angle (15°) and the Smoluchowski approximation for aqueous solutions were applied. Data collection was performed at 25 °C. Two hundred runs of measurements were performed for each sample to obtain the mean zeta potential. Individual samples were measured 5 times.

### 2.5. Transmission Electron Microscopy

EVs were diluted 1:10 with DPBS, pipetted onto a glow-discharged carbon-coated copper grid, and incubated for 10 min. Next, the grid was washed in 3 droplets of double-distilled water and immersed for 3–5 s in a drop of 2% aqueous uranyl acetate for negative staining, as previously described [26]. Afterwards, uranyl acetate remnants were blotted with filter paper and samples were allowed to dry. EVs were imaged with a Jeol 1400 transmission electron microscope (accelerating voltage 120 kV, Jeol, Tokyo, Japan).

### 2.6. EV Fluorophore Labeling

EVs were stained with the ExoGlow Membrane EV Labeling Kit (EXOGM600A-1, System Biosciences, Newark, CA, USA) according to the manufacturer’s instructions. Briefly, 2 µL labeling dye and 12 µL reaction buffer were mixed and added to 80–100 µg EV protein equivalents. The labeling reaction was performed for 30 min protected from light. For removal of free dye, labeled EVs were loaded onto a PD SpinTrap G-25 column (28918004, Cytiva, Marlborough, MA, USA) and centrifuged at 800× *g* for 1 min. The flow-through containing purified labeled EVs was aliquoted and stored at −80 °C.

### 2.7. Flow Cytometric Quantification of EV Uptake

Colonic organoids were passaged as previously described. Fifteen microliters of organoid fragments resuspended in Matrigel were seeded into V-bottom 96-well plates and allowed to recover overnight. The following day, organoids were incubated with 0.5, 1, or 5 µg of Exo-labeled EVs derived from LR or LP for 6 h. After incubation, organoids were released from Matrigel using Cell Recovery Solution (354253, Corning, NY, USA), briefly digested with Trypsin-EDTA, mechanically dissociated to generate a single-cell suspension, and fixed with 4% paraformaldehyde. Samples were analyzed using an Attune flow cytometer (Attune NxT, Thermo Fisher Scientific, Waltham, MA, USA) and Attune Cytometric Software (v5.3.0). For re-gating and further analysis, raw FCS files were imported into FlowLogic software (v8.7, Inivai Technologies, Mentone, Australia).

### 2.8. Viability Assay

EV treatment of colonic organoids for viability analysis followed the same protocol as described in “Flow cytometry-based quantification of EV uptake”, with the exception that native EVs were used. Organoid viability was assessed by quantifying intracellular ATP levels using the CellTiter-Glo 3D Cell Viability Assay (G9681, Promega, Madison, WI, USA) according to the manufacturer’s instructions. Luminescence signals were recorded using a Tecan Infinite 200Pro microplate reader (Tecan Life Sciences, Männedorf, Switzerland) and analyzed with the Tecan i-control software (v2.0.10.0).

### 2.9. EdU Assay

DNA synthesis was evaluated using the Click-it EdU Alexa Fluor 647 Imaging Kit (C10340, Invitrogen, Waltham, MA, USA). Colonic organoid-derived monolayers were treated with 0.5, 1, or 5 µg of native EVs from either LR or LP strains for a total of 6 h. Staining with 5-ethynyl-2-deoxyuridine (EdU) reagent (10 µM) was performed after 3 h of EV exposure to label newly synthesized DNA. All subsequent steps were performed according to the manufacturer’s instructions.

### 2.10. Apoptosis Assay

To evaluate apoptosis in EV-treated colonic organoids, the CellEvent Caspase-3/7 Green Flow Cytometry Kit (C10427, Invitrogen, Waltham, MA, USA) was used with minor protocol modifications. Organoid processing and EV treatment were the same as for the viability assay. Briefly, dissociated single cells from organoids were resuspended in 1 mL of DPBS and transferred to flow cytometry tubes. Each sample was incubated with 1 µL of CellEvent Caspase-3/7 Green Detection Reagent (final concentration: 500 nM) for 45 min at 37 °C. During the final 10 min, 1 µL of SYTOX AADvanced Dead Cell Stain (final concentration: 1 µM) was added. Samples were analyzed using an Attune NxT flow cytometer (ThermoFisher Scientific, Waltham, MA, USA) with Attune Cytometric Software (v5.3.0). For re-gating and advanced analysis, raw FCS files were processed using FlowLogic software (v8.7, Inivai Technologies, Mentone, Australia).

### 2.11. Endo-Lysosomal Colocalization Assay

Intracellular trafficking of EVs was studied employing a pulse-chase assay as described by [27,28]. Colonic monolayers cultured in 8-well chamber slides (94.6190.802, Sarstedt, Nümbrecht, Germany) were cooled down on ice to 0–4 °C, incubated with 5 µg of Exo-labeled EVs for 30 min (“pulse”), and then rinsed with warm EV-free medium to remove unbound labeled EVs. After cultivation at 37 °C, 5% CO_2_ was maintained again; EV internalization was monitored by live-cell imaging at 10-min intervals (“chase”). Sorting of internalized EVs into acidic endo-lysosomal compartments was examined by pre-staining monolayers with LysoTracker Blue (LT, L7525, Invitrogen, Waltham, MA, USA) according to the manufacturer’s protocol. To quantify co-localization of Exo-labeled with endo-lysosomal acidic compartments, integrated density of Exo-positive LT-positive puncta was quantified. Briefly, original image files were split into two channels (Exo, LT), duplicated, and appropriate cells were selected. After applying a median filter (6 px), filtered images were subtracted from the original images and the threshold was adjusted by Otsu’s method for each channel. All puncta exceeding the threshold were selected, saved as regions of interest (ROI), and the spatial intersection between the ROIs of the Exo- and LT channels was determined. The resulting overlap ROI was applied to the filtered image of the Exo channel to determine integrated density.

### 2.12. LTA Blocking Experiments

The role of lipoteichoic acid (LTA)-mediated EV uptake was assessed by blocking presumed ligand–receptor interactions using a monoclonal antibody targeting the conserved LTA epitope of Gram-positive bacteria (ab267414, Abcam, Cambridge, UK) or the corresponding isotype antibody (Monoclonal mouse IgG1, NBP2-21942, BioTechne, Minneapolis, MN, USA), applied at concentrations of 10 µg/mL, 1 µg/mL, 0.1 µg/mL, and 0.01 µg/mL, respectively. For reverse inhibition by saturating LTA binding sites on the epithelium, LTA extracted from LR bacteria was used. To avoid cytotoxic damage, prevent signal oversaturation from Exo-labeled EVs, and enable detection of subtle inhibitor-dependent effects, a 1:100 dilution (0.05 µg) of the initial EV input dose of 5 µg and a shortened incubation period were chosen. Isolation of LTA was performed as previously described by Kho and Meredith [29]. Briefly, 4 L of LR bacterial suspension were harvested by centrifugation (6000× *g*, 10 min) and the pellet was lysed with glass beads (G1145, Sigma-Aldrich, St. Louis, MO, USA) using a Precellys Evolution (Bertin Technologies, Montigny-le-Bretonneux, France). Following high-speed centrifugation (30,000× *g*, 4 °C, 45 min), pelletized bacterial membranes were mixed with 1 volume of *n*-butanol and incubated for 45 min under agitation at 37° C (Thermomixer Compact, Eppendorf, Hamburg, Germany). Following phase separation by centrifugation, the aqueous phase containing LTA was collected, lyophilized twice, and reconstituted with DPBS to a concentration of 100 mg/mL. Successful extraction of LTA was verified by ^1^H Nuclear Magnetic Resonance (NMR) spectroscopy (Bruker Avance Neo 600 MHz, Billerica, MA, USA; software TopSpin v4.4.1 and MestReNova v14.2.0-26256).

Confocal images were acquired using either a Zeiss LSM710 confocal microscope with a 63×/NA = 1.0 water immersion objective or a Leica SP8 system (Leica Microsystems, Wetzlar, Germany) equipped with a 40×/NA = 1.3 oil immersion objective. For live-cell imaging, cells were maintained at 37 °C under normoxic conditions in a stage-top incubator throughout the acquisition process. Fluorescence images were visualized and quantitatively analyzed using Fiji (ImageJ, v1.54p) [30].

### 2.13. Analysis of Long-Chain Fatty Acids

All EV samples were obtained from 500 mL of culture supernatant with OD_600_ values ranging between 4.0 and 5.0 to normalize for EV biomass. Sixty µL of each sample was evaporated and mixed with 1 volume of *n*-hexane. Twenty-five µL of each sample was used for further analysis. The samples were derivatized with 600 µL of methanolic HCl 3 N (85 °C) for 45 min. Subsequently, samples were analyzed on a LECO TOF Pegasus mass spectrometer (LECO corporation, St. Joseph, MI, USA). For gas chromatography/mass spectrometry (GC/MS) analysis, a BTX-70 column (SGE Analytical Science, Melbourne, Australia; 25 m, inner diameter of 0.22 mm, path length of 0.25 µm). 1 µL of sample was injected splitless with helium as the carrier gas at a flow rate of 1.3 mL/min. A Supelco 37 Component FAME MIX in dichloromethane was used as a standard. Data were analyzed using the LECO Chroma TOF software v4.72.0.0 (*m*/*z* 74). 

### 2.14. Western Blot

EVs, bacterial cells, and colonic organoids were lysed with NP40 lysis buffer supplemented with PhosSTOP and cOmplete Mini protease inhibitor cocktail (both from Roche) and denatured with 6× Laemmli buffer. Depending on the expected band size, electrophoresis was performed on 10 or 12% SDS-polyacrylamide gels in a Mini-PROTEAN Tetra Cell (Bio-Rad, Hercules, CA, USA). After dry-blotting using the iBlot Gel Transfer System (Invitrogen, Waltham, MA, USA), the following primary and secondary antibodies were used: Gram-positive bacteria antibody (1:1000; ab267414, Abcam), BiP/Grp78 (1:1000; 3177, Cell Signaling Technology, Danvers, MA, USA), CHOP (1:1000, 2895, Cell Signaling Technology, Danvers, MA, USA), phospho-eIF2α (Ser51) (1:1000, 9721, Cell Signaling Technology, Danvers, MA, USA), eIF2α (1:1000, 5324, Cell Signaling Technology, Danvers, MA, USA), and beta-actin (1:1000, 1978, Sigma-Aldrich, St. Louis, MO, USA), anti-mouse IgG HRP-linked secondary antibody (1:5000; NA931V, Amersham, Little Chalfont, UK), and anti-rabbit IgG HRP-linked secondary antibody (1:5000, NA934V, Amersham, Little Chalfont, UK). Bands were visualized by chemiluminescence using the SuperSignal West Dura Extended Duration Substrate (34076, ThermoFisher Scientific, Waltham, MA, USA) and Imaging system Fusion Solo X (Vilber Lourmat, Collégien, France). Band intensity was normalized to either the intensity of Coomassie Brilliant Blue staining (bacterial EVs and bacterial cells) or β-actin bands (colonic organoids). To generate positive controls for unfolded protein response activation, colonic organoids were treated with tunicamycin (T7765, Sigma-Aldrich, St. Louis, MO, USA, 0.5 µg/mL).

### 2.15. Proteomic Mass Spectrometry

#### Preparation of Bacterial Cells and EV Samples

Solid bacterial cell pellets were resuspended in 500 µL of urea/thiourea buffer and subjected to 3 cycles of freeze–thaw using liquid nitrogen. Subsequently, 3 cycles of homogenization were performed using a FastPrep system (5.5 m/s for 60 s), with a 5 min resting period between each cycle. The sample was incubated at 75 °C for 10 min at 1400 rpm in a thermomixer. After incubation, the sample was centrifuged at full speed for 10 min at 4 °C, and the supernatant was transferred into a fresh tube. Protein concentration was determined using the Bradford assay. A total of 100 µg of protein, adjusted to a final volume of 30 µL, was loaded onto a FASP column, and the FASP protocol was followed for protein digestion using trypsin/Lys-C endopeptidase. Peptide purification was performed using SOLA µ solid-phase extraction. The purified peptides were resuspended in 17 µL of 0.1% formic acid, and 2 µL was used for quality control on a Nanodrop spectrophotometer. Finally, the peptide concentration of the samples was adjusted to 0.2 µg/µL for subsequent analysis.

EVs solubilized in 100 µL DPBS were denatured with 10% SDS and incubated for 10 min at 70 °C with shaking at 1400 rpm. Subsequently, 25 µL of the lysate was mixed with 5 µL of 5× loading buffer. The mixture was then incubated for 3 min at 95 °C while shaking at 1400 rpm. One-dimensional gel electrophoresis was performed, allowing the gel to run until the entire sample had entered the separating gel. The entire protein gel was excised and cut into small pieces for further processing. An in-gel trypsin/lys-C digestion was conducted to prepare peptides for downstream analysis. The digested peptides were purified using SOLA µ columns. The resulting solution was resuspended in 17 µL of 0.1% formic acid, with 2 µL used for Nanodrop measurements to determine peptide concentration. Finally, the samples were diluted and adjusted to a protein concentration of 0.2 µg/µL for further analysis.

### 2.16. Mass Spectrometry

A nano-high-performance liquid chromatography (HPLC) system (UltiMate 3000 RSLCnano system, ThermoFisher Scientific, Waltham, MA, USA) was used to separate the cleaved peptide lysates. The system was connected to a Q Exactive HF Orbitrap mass spectrometry (LC-MS/MS) system (ThermoFisher Scientific, Waltham, MA, USA) equipped with the nanoelectrospray ion source TriversaNanoMate (Advion, Ithaca, NY, USA). The database search was performed with Proteome Discoverer (v2.5.0.8, ThermoFisher Scientific) applying the SEQUEST-HT algorithm. The proteomes of *Limosilactobacillus reuteri* strain DSM 20016 (Uniprot ID: UP000001991) and *Lactiplantibacillus plantarum* strain WCFS1 (Uniprot ID: UP000000432), being the most closely related strain to LP with the most comprehensive protein annotation, were used as references. The precursor mass tolerance of the MS was set to 10 ppm, and the fragment mass tolerance of the MS/MS was 0.02 Da. Carbamidomethylation of cysteine was considered fixed, and oxidation of methionine was set as a dynamic modification. Enzyme specificity was set to trypsin with up to two missed cleavages, using 10 ppm peptide ions and 0.02 Da MS/MS tolerances. Only rank-one peptides with a Percolator-estimated false discovery rate < 1% were accepted as identified. Proteins with two or more missing values across three replicates, as well as those labeled as contaminants, were filtered out during data preprocessing.

### 2.17. Preparation of EV-Treated Colonic Organoids

Colonic organoids were cultivated and passaged as previously described. Fifteen microliters of organoid fragments resuspended in Matrigel were seeded into V-bottom 96-well plates and, following overnight recovery, incubated with 5 µg native EVs from LR or LP for 6 h. Organoids were released from Matrigel using Cell Recovery Solution (354253, Corning, NY, USA), washed with DPBS, and frozen as pellets at −80 °C. The organoids were resuspended in 100 µL of UT buffer and subjected to 3 freeze–thaw cycles using liquid nitrogen. Subsequently, the samples were incubated in an ultrasonication bath for 5 min. After incubation, they were centrifuged at maximum speed for 10 min at 4 °C, and the resulting supernatant was transferred to a fresh tube. Protein digestion was carried out using trypsin/Lys-C endopeptidase with overnight incubation at 37 °C. Peptides were purified using SOLA µ solid-phase extraction, then resuspended in 17 µL of 0.1% formic acid. A 2 µL aliquot was assessed for quality using a Nanodrop spectrophotometer. Finally, peptide concentrations were adjusted to 0.2 µg/µL for downstream analysis.

### 2.18. Mass Spectrometry

For each nanoLC-MS measurement, 1 µg of peptides was injected into a Vanquish Neo nanoHPLC system (ThermoFisher Scientific, Waltham, MA, USA). Initial trapping occurred on a C18-reverse phase trapping column (Acclaim PepMap 100, 75 μm × 2 cm, particle size 3 μm, nanoViper, ThermoFisher Scientific, Waltham, MA, USA), followed by subsequent separation on a C18 reverse phase analytical column (Double nanoViper PepMap Neo, 75 μm × 150 mm, particle size 2 μm, ThermoFisher Scientific, Waltham, MA, USA). The separation utilized a two-step gradient employing mobile phases A (0.01% formic acid in H_2_O) and B (80% acetonitrile in H_2_O with 0.01% formic acid). During the first 65 min, the proportion of mobile phase B increased from 4% to 30%, followed by a subsequent 30 min period during which the proportion of mobile phase B rose from 30% to 55%. The flow rate was maintained at 300 nL per minute throughout the separation process. The eluted peptides underwent ionization using a Nanospray Flex Ion Source (ThermoFisher Scientific, Waltham, MA, USA) and were subsequently detected by an Orbitrap Exploris 480 mass spectrometer (ThermoFisher Scientific, Waltham, MA, USA) operating in data-dependent acquisition (DDA) mode.

The database search was performed with Proteome Discoverer (v2.5.0.8, ThermoFisher Scientific, Waltham, MA, USA) against the UniProt reference proteome of *Mus musculus* (UP000000589), employing the SEQUEST-HT algorithm. The precursor mass tolerance of the MS was set to 10 ppm, and the fragment mass tolerance of the MS/MS was 0.02 Da. Carbamidomethylation of cysteine was considered fixed, and oxidation of methionine was set as a dynamic modification. Enzyme specificity was set to trypsin with up to two missed cleavages. Only rank-one peptides with a Percolator-estimated false discovery rate < 1% were accepted as identified. Following log2-transformation and median-normalization, technical replicates were averaged within each of three biological replicates (*n* = 3). Proteins with <2 complete paired biological replicates or ≥2 missing values within either condition, as well as those labeled as contaminants, were excluded. For proteins with one remaining missing value, no imputation was performed.

### 2.19. Quantification and Statistical Analysis

All statistical analysis was performed using R, specifically using the packages tidyverse and BiocManager [31,32]. Results were expressed as mean values and their respective standard deviation (SD). All graphical plots, including Principal Component Analysis (PCA), Venn diagram, sample-to-sample distance matrix, pathway coverage analysis, and volcano plot, were created using the R packages tidyverse, pheatmap, clusterProfiler (v4.20.0 [33]), and Venn diagram. For murine proteomic datasets, differential protein abundance analysis was performed using the R package limma [34], followed by empirical Bayes moderation with intensity-dependent variance modeling. Gene Ontology (GO) enrichment results were generated using the web server-based DAVID Bioinformatics Resources [35]. UniProt accession numbers were mapped to *Mus musculus* Entrez Gene IDs using org.Mm.eg.db (v3.23.0). KEGG overrepresentation analysis was performed using the R package enrichKEGG. FASTA sequence-based prediction of the subcellular localization of bacterial cell and EV proteins was performed using PSORTb v3.0.3 [36]. Putative lipoproteins were validated using the PRED-LIPO database [37].

## 3. Results

### 3.1. LR and LP EVs Exhibit Similar Morphology and Size Distribution but Different Zeta-Potential

**Figure 1:**
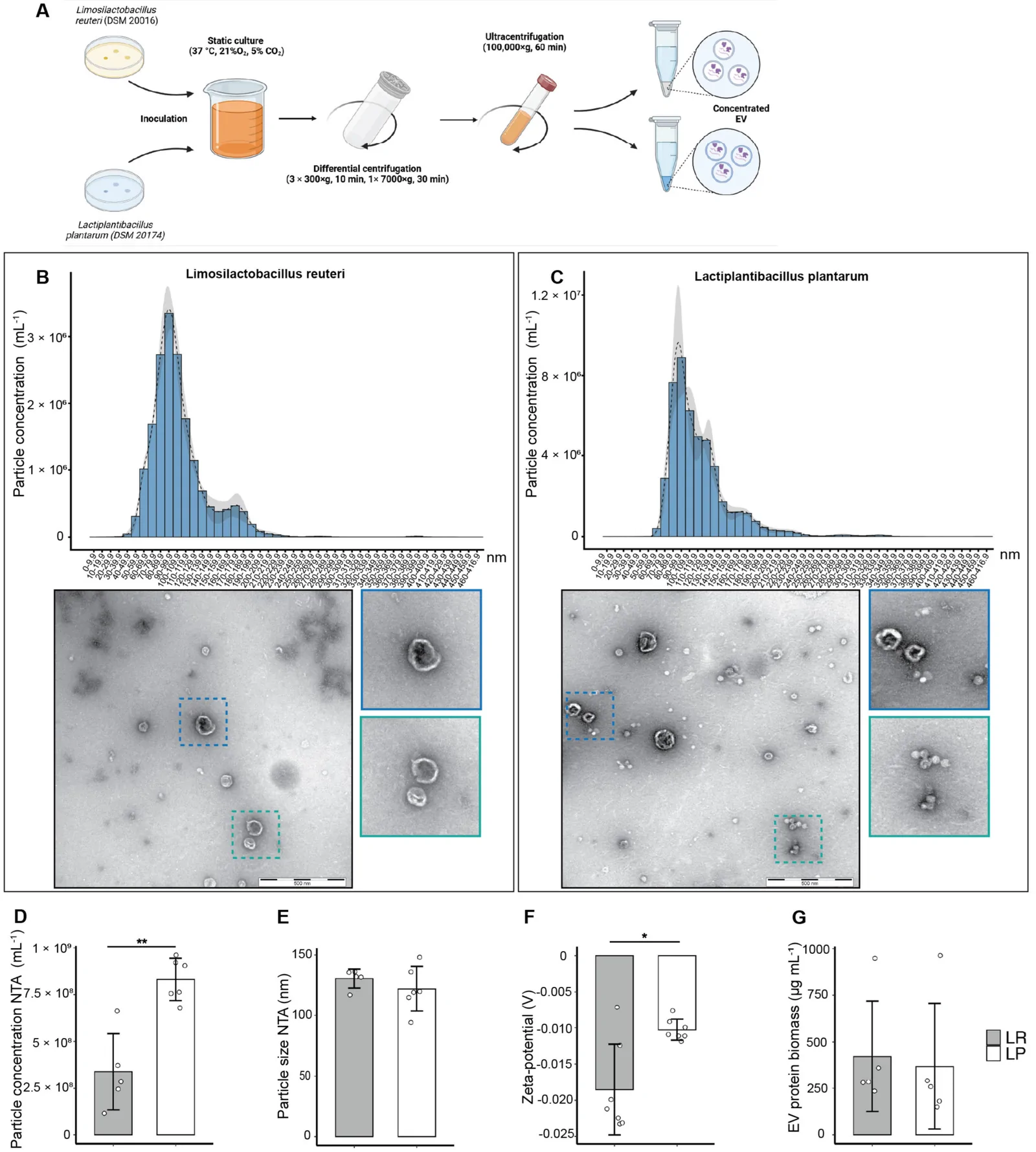
Characterization of EVs from *Limosilactobacillus reuteri* (LR) and *Lactiplantibacillus plantarum* (LP). (**A**) Illustration of the technical workflow for bacterial EV isolation. The scheme of the experimental set-up was created with Biorender.com. (**B**,**C**) Nanoparticle tracking analysis (NTA) showed unimodal size distribution curves for LR (**B**) and LP EVs (**C**). Curves are overlaid with bars representing the mean of binned particle sizes; the gray shaded area displays SD (**top**). Negative contrast-enhanced transmission electron microscopy (TEM) revealed spherical EVs enclosed by a lipid membrane (whitish rim); scale bar: 500 nm. Small insets on the left show enlarged views of selected LR and LP EVs (**bottom**). (**D**) LP bacteria produced a significantly higher EV particle concentration compared to LR bacteria as determined by NTA (*n* = 6 independent EV preparations per species; NTA data for one LR preparation was unavailable due to a technical measurement failure). (**E**) No significant difference in mean NTA particle size was observed between LR and LP EVs. (**F**) Zeta-potential readings using electrophoretic light scattering (ELS) were obtained from EVs solubilized in 5% dextrose/1 mM NaCl at 25 °C, showing a significantly lower (i.e., more negative) surface charge in LR EVs compared with LP EVs (*n* = 7 independent EV preparations per species). (**G**) Protein biomass concentrations (in µg/mL) as determined by the Bradford assay did not differ between LR and LP EVs (*n* = 6 independent EV preparations per species). Statistical comparisons were performed using Welch’s corrected *t*-test (*p*-value: ns > 0.05; * < 0.05; ** < 0.01). Bars indicate mean ± SD.

We began by assessing whether the selected bacterial species LR and LP produced EVs in sufficient quantities for reproducible mass spectrometry and cell culture experiments. To ensure EVs were isolated from viable bacterial cells, supernatants were collected from bacterial suspensions with an OD_600_ of 4.0–5.0, as previously determined by growth curve analysis (Figure S1). EVs were subsequently concentrated from the culture supernatant by ultracentrifugation, following a protocol adapted from Dean et al. [38]. Major steps of the experimental workflow are illustrated in Figure 1A.

To assess the quality of the EV preparations, we performed Nanoparticle tracking analysis (NTA) (Figure 1B,C; top) and transmission electron microscopy (TEM) (Figure 1B,C; bottom) to quantify particle concentration, determine size distribution, and confirm the presence of vesicular structures. Negative-stain contrast enhancement revealed predominantly spherical particles, some of which exhibited a cup-shaped morphology. A relevant interference with particles originating from medium components was previously excluded (Figure S2A).

LP-derived EVs exhibited a significantly higher mean particle concentration than those from LR (Figure 1D). In line with previous reports [38,39], both EV populations displayed a right-skewed unimodal size distribution, with mean diameters of 131.0 ± 7.82 nm for LR EVs and 122.0 ± 18.6 nm for LP EVs, respectively (Figure 1E). As an accessory approach, we performed TEM-based measurements of single-vesicle diameters from a representative EV preparation that did not differ between LR and LP either. The apparently smaller EV diameters observed by TEM are likely attributable to dehydration artifacts as a consequence of sample preparation. We performed electrophoretic light scattering (ELS) under isosmotic, ultra-low salt conditions to minimize ionic interference with zeta-potential measurements. LR EVs exhibited a significantly lower (i.e., more negative) zeta-potential compared to LP EVs (−0.0185 ± 0.0062 V vs. −0.0103 ± 0.0014 V), indicating a difference in net surface charge between the two EV types (Figure 1F and Figure S3A). Corresponding conductivity and electrophoretic mobility readings are available in the supplementary files (Figure S3B,C). Protein biomass concentrations and particle-to-protein ratios obtained from LR and LP EV preparations were similar (Figure 1G and Figure S2B). A contamination of isolated EVs with living bacteria was excluded (Figure S2C).

### 3.2. LR and LP EVs Are Equipped with a Similar Composition of Long-Chain Fatty Acids and Lipoteichoic Acid

**Figure 2:**
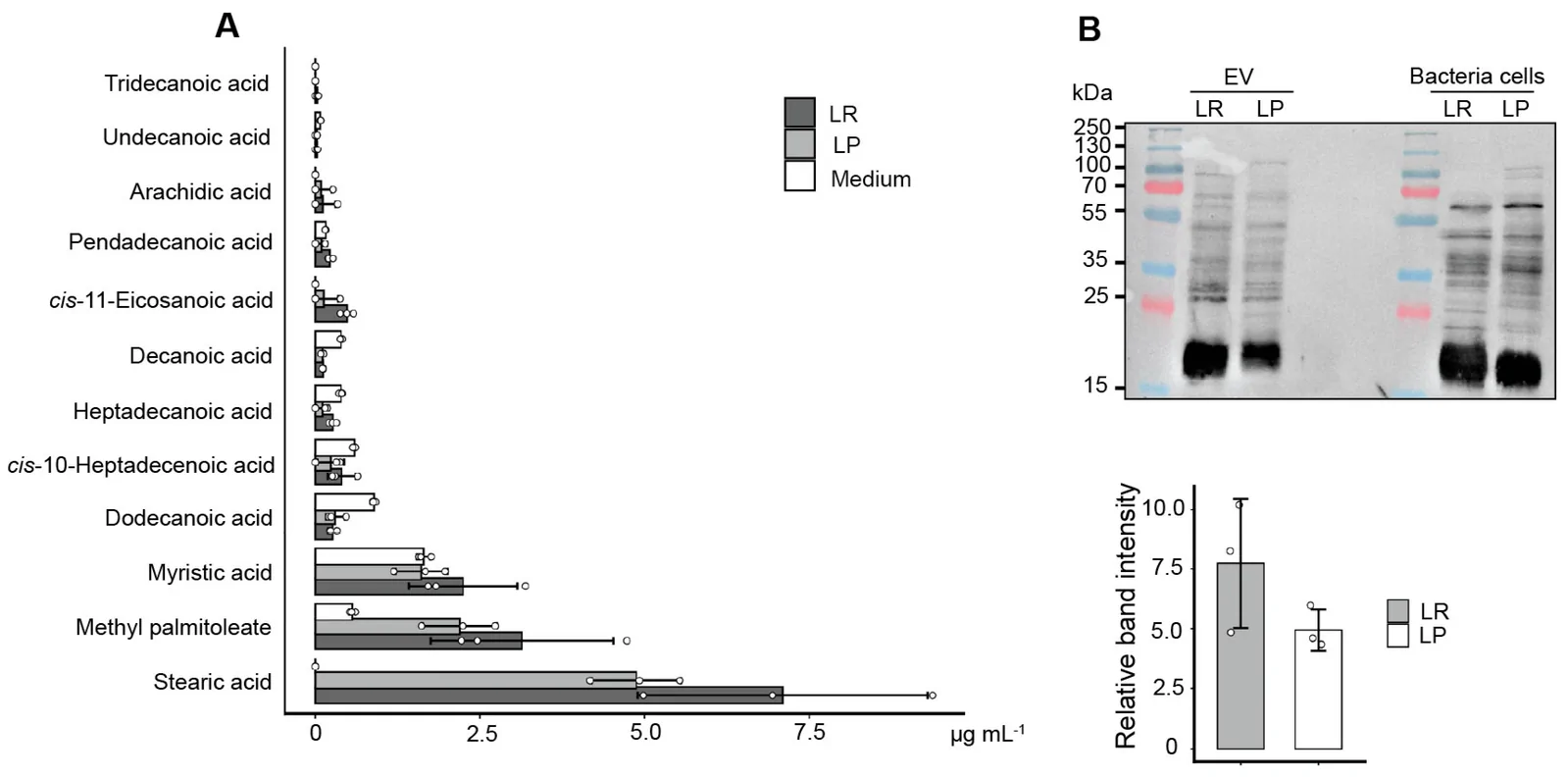
LR and LP EVs contain a similar spectrum of long-chain fatty acids and lipoteichoic acid. (**A**) Gas chromatography/mass spectrometry (GC-MS) analysis of long-chain fatty acids (LCFA) extracted from LR and LP EV preparations (*n* = 3 independent replicates). Medium control refers to non-inoculated concentrated MRS medium. LCFA concentrations (mean ± SD) are reported in µg/mL (x-axis). (**B**) Western blot for detection of EV-bound lipoteichoic acid (LTA) normalized to total protein content through Coomassie brilliant blue staining (*n* = 3 independent EV preparations). EV samples were washed with DPBS and underwent repeat ultracentrifugation prior to denaturation and loading 2.5 µg EV protein equivalent onto the gel. As a positive control, whole bacterial cells lysed with NP-40 buffer/0.5% Triton-X, followed by mechanical disruption in an ultrasonic water bath were used (**top**). Densitometry indicated a slightly higher amount of LR EV-bound LTA (**bottom**).

We employed GC/MS analysis of vesicle membrane preparations to assess whether lipid signatures of the vesicle membranes differ by the parental bacterial species (Figure 2A). Stearic acid (C18:0), methyl palmitoleate (C17:1(9Z)), and myristic acid (C14:0) were the most prevalent saturated long-chain fatty acids (LCFA) encountered in EV preparations from either species. Compared with non-inoculated medium, stearic acid was the only LCFA consistently detected at relevant concentrations in EV samples, indicating that several other LCFAs are naturally present in MRS broth. In addition, trace amounts of the monounsaturated LCFA *cis*-11-eicosenoic acid (C20:1 (11Z)) and the saturated LCFAs arachidic acid (C20:0) and tridecanoic acid (C13:0) were exclusively detected in EV preparations (LCFAs listed in descending order; Table S1). These findings support the notion that stearic acid, and to a much lesser extent *cis*-11-eicosenoic acid, arachidic acid, and tridecanoic acid, are components of the membrane leaflets of LR- and LP-derived EVs.

A few studies report the presence of lipoteichoic acid (LTA) incorporated in the EVs derived from Gram-positive bacteria [13,14]. Polyanionic type 1 LTA functions as a linker tethering the Gram-positive cell wall to the cytoplasmic plasma membrane and is likely passed on to the nascent EV membrane. Given the previously reported difference in zeta-potential between LR and LP EVs, we quantified EV-bound LTA normalized to total protein content, which was detectable but did not differ significantly between the two species (Figure 2B).

### 3.3. LR and LP EVs Share a Similar Proteome with Their Parental Bacteria and Contain Lipoproteins

To resolve the protein cargo of the EVs and their parental bacteria, we employed proteome mass spectrometry. The list of identified proteins and protein abundances is included in Table S4. In a first exploratory step, we performed Coomassie brilliant blue staining of EVs and whole bacterial cell proteins separated by SDS-PAGE (Figure S4A). The resulting arrangement of bands allowed us to optically distinguish EVs from bacterial cells. On bacteria cell level, band patterns appeared unique to each species, but no obvious demarcation at the EV level was noted. Next, we set out to characterize the proteome of EVs and their parental bacterial cells by LC-MS/MS. In total, 3224 proteins were identified, of which 1218, 607, 960, and 439 proteins were detected in LR bacteria, LP bacteria, LR EVs, and LP EVs, respectively. A limited number of 361, 245, 103, and 77 proteins were unique to LR bacteria, LP bacteria, LR EVs, and LP EVs, respectively (Figure S4B). Discrepancies in the absolute numbers of identified whole-cell and EV-associated proteins may be influenced by the necessity to apply different extraction protocols accounting for varying protein content and complexity of the input material. Principal component analysis (PCA) showed a distinct separation of samples both by species and sample type, with the latter being the main source of variance (PC1: 71.3%) (Figure S4C). A sample-to-sample distance matrix revealed the greatest difference between LR EVs and LP bacteria, followed by LP EVs versus LP bacteria and LR EVs and LR bacteria, respectively (Figure S4D).

**Figure 3:**
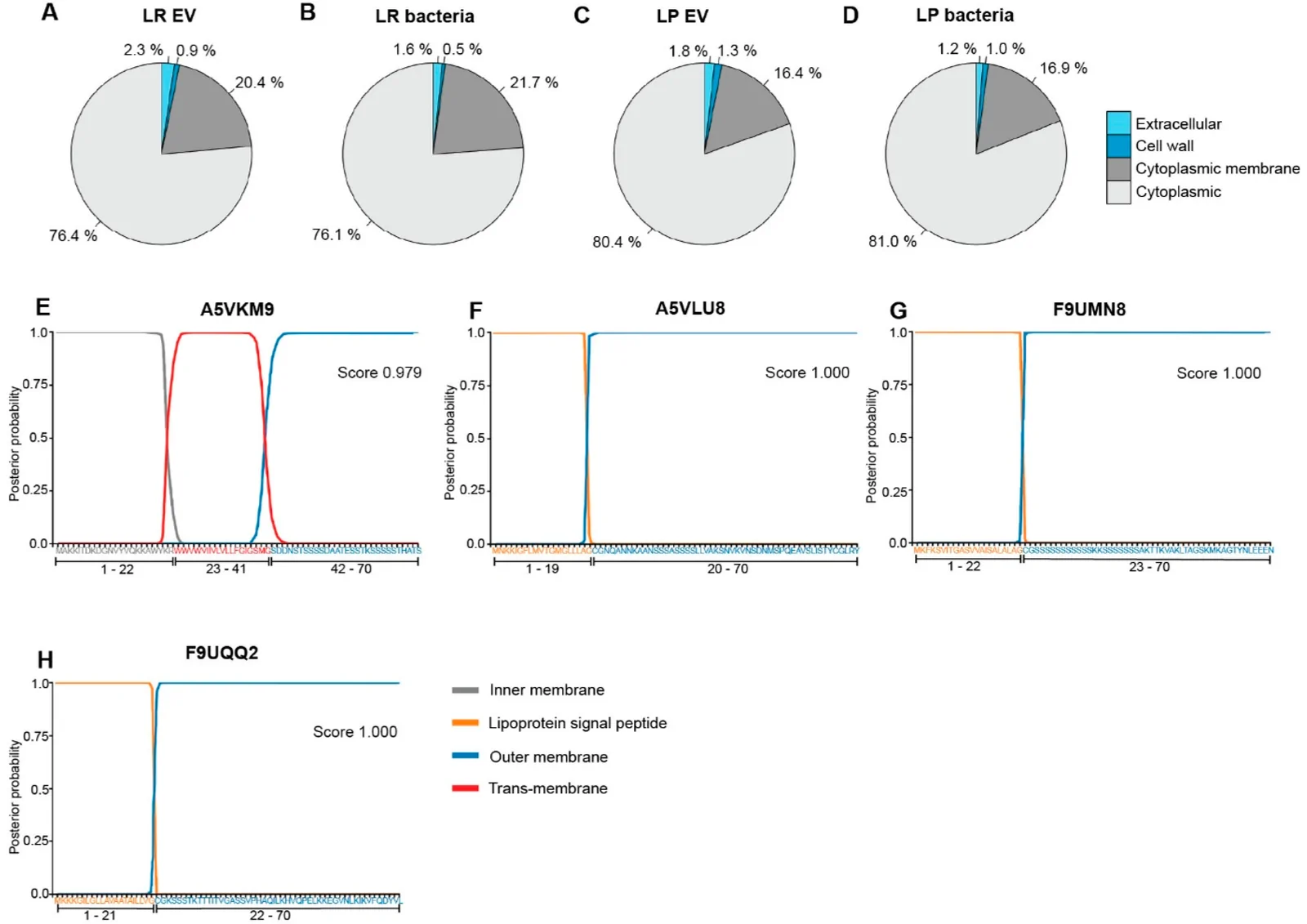
EVs released from LR and LP bacteria share a similar distribution of compartmentalized proteins (**A**–**D**). Pie charts illustrating the predicted subcellular localization of proteins extracted from LR EVs (**A**), LR bacterial cells (**B**), LP EVs (**C**), and LP bacterial cells (**D**) using PSORTb. Proteins allocated to the domains “Extracellular”, “Cytoplasmic membrane”, “Cytoplasm”, and “Cell wall” are displayed as percentage values. (**E**–**H**). Structural prediction of lipoprotein signal peptides for four selected proteins (identified by UniProt accession numbers) using the PRED-LIPO web-based tool: A5VKM9 (**E**) and A5VLU8 (**F**) from LR, F9UMN8 (**G**) and F9UQQ2 (**H**) from LP. Topology plots were adapted from web server-generated graphical output using WebPlotDigitizer v5.2 (https://automeris.io, accessed on 1 August 2026).

Employing PSORTb, we predicted the subcellular localization of EV and bacterial proteins to filter for proteins likely to possess extracellular domains or secretory signal peptides and thus be capable of interacting with the host epithelium (Figure 3A–D). After excluding non-classifiable proteins, most proteins were predicted to be localized in the cytoplasm. By contrast, only a small fraction of proteins was predicted to be exported to the extracellular milieu, or to associate with the cell wall. Among the ten most abundant proteins predicted to localize to the cytoplasmic membrane (Table S2) and the extracellular compartment (Table S3), we identified four candidate proteins in LR- and LP-derived EVs (two per species), with features consistent with lipoproteins (Lpps). To validate that prediction, structural inference was cross-verified using the PRED-LIPO database, which confirmed three proteins as Lpps with maximum confidence scores (1.00; two in LP EVs and one in LR EVs), while one additional protein from LR EVs exhibited putative lipoprotein characteristics with lower confidence (score 0.979) (Figure 3E–H) [37].

**Figure 4:**
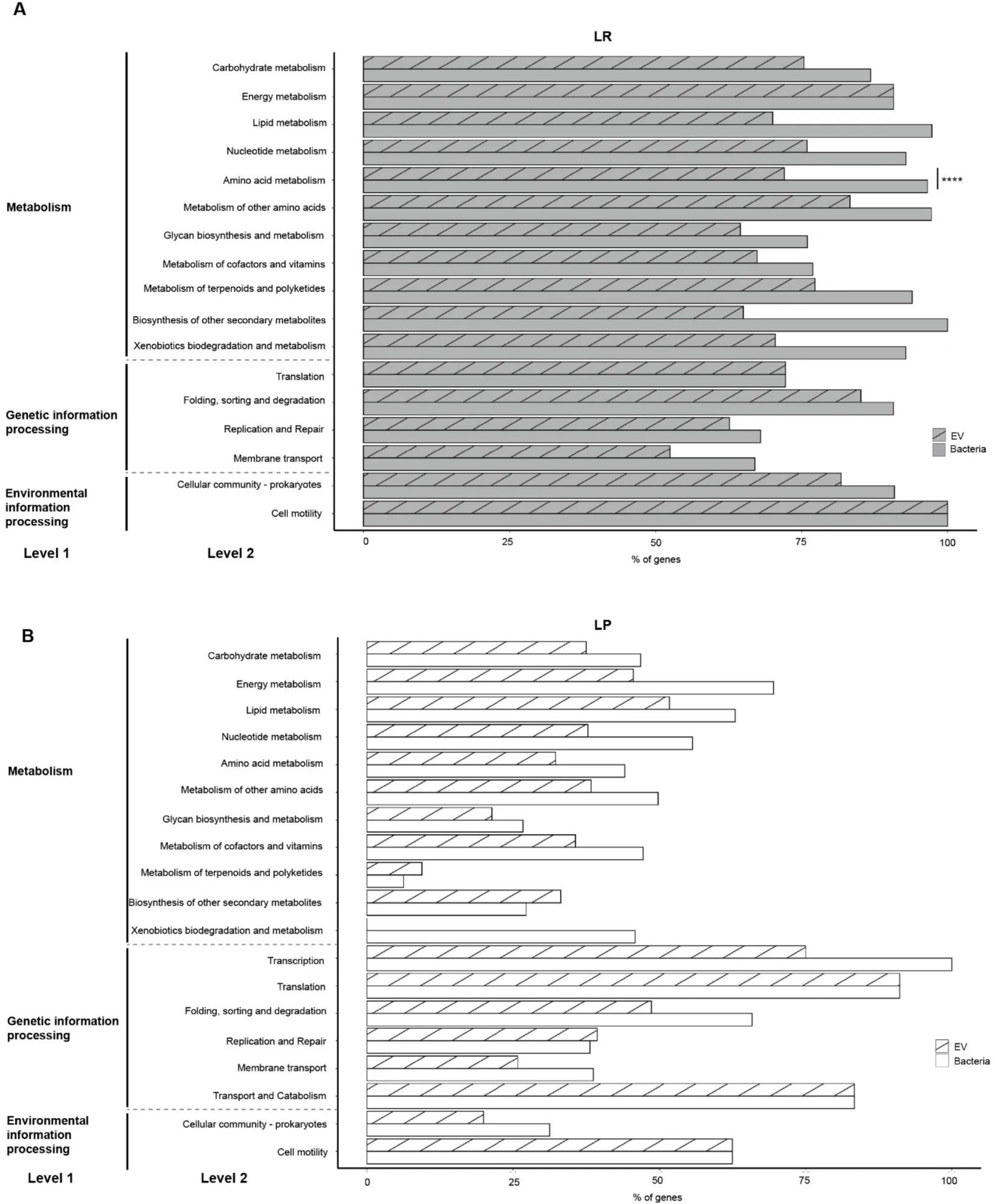
Proteomic cargo analysis of EVs and corresponding bacterial cells failed to identify determinants of differential EV uptake. (**A**,**B**). Mean pathway coverage of prokaryotic Kyoto Encyclopedia of Genes and Genomes (KEGG) pathway maps subsumed under a given level 2 functional category was compared between bacterial cells and EVs for LR (**A**) and LP (**B**), respectively (*n* = 3 independent replicates per species and sample type). Pathway coverage refers to the percentage of detected genes related to the total number of genes annotated to a given KEGG pathway. All pathway entries mapped to LR (lre, entry T00535) and LP (lpl, entry T00115) were retrieved from the KEGG database and each Uniprot ID was matched with its corresponding gene ID. Enrichment of proteins included in a given KEGG pathway was verified by two-sided Fisher’s Exact test (*p*-value < 0.05). Statistically significant differences in mean pathway coverage were assessed species-wise with Student’s *t*-test (*p*-value: **** < 0.0001).

Additionally, we performed pathway coverage analysis to compare species-specific representations of bacterial cells and EVs across KEGG functional hierarchy levels 1 and 2. LR bacterial cells exhibited markedly higher coverage rates of level 2 categories, spanning a broad array of core metabolic pathways implicated in amino acid, carbohydrate, glycan, nucleotide, and lipid turnover (Figure 4A). To a lesser degree for most categories, the same pattern was mirrored by the EV proteome. Only a limited number of categories, including pathways linked to ribosomal protein synthesis and transcription, transport and catabolism, and cell motility, surpassed a 50% coverage threshold in LP EVs and whole bacterial cell proteomes. Pathways pertaining to biosynthesis and metabolism of terpenoids, polyketides, and other secondary metabolites were modestly enriched in LP EVs, whereas all other functional categories remained underrepresented in EVs relative to the LP whole bacterial cell proteome. Contrary to expectations, factual proteomic coverage did not correlate with the larger genome size of LP species, which may be explained by differential gene expression influenced by environmental conditions as well as incomplete matching with the selected reference proteome of *Lactiplantibacillus plantarum* strain WCFS1 [40,41] (Figure 4B).

### 3.4. LR and LP EVs Differ in Their Uptake Efficiency into Colonic Epithelium and Interfere with Host Cell Homeostasis

Next, we set out to assess the impact of EVs on primary colonic epithelium. Colonic organoids were either maintained in basement membrane matrix or grown as a single cell-derived monolayer, with either basolateral or apical membranes everted to the surrounding medium, respectively. Based on published reports estimating the total biomass of secreted gut microbiota EVs to be roughly 320 mg, we inferred the amount of EV per mucosal surface area of the colon (2 m^2^), resulting in a density of 16 µg/cm^2^ [42,43]. Following adjustment to the average growth area of the wells, we selected an initial amount of 5 µg EVs to set a benchmark for later dose refinements. Importantly, this system does not claim to reflect physiological reality, as calculations are a mere approximation of luminal exposure and EVs originate from a single species. The incubation period was restricted to 6 h after extended time spans up to 24 h had indicated unrelenting EV uptake into cells, resulting in overload and ultimately macroscopic cell disruption.

**Figure 5:**
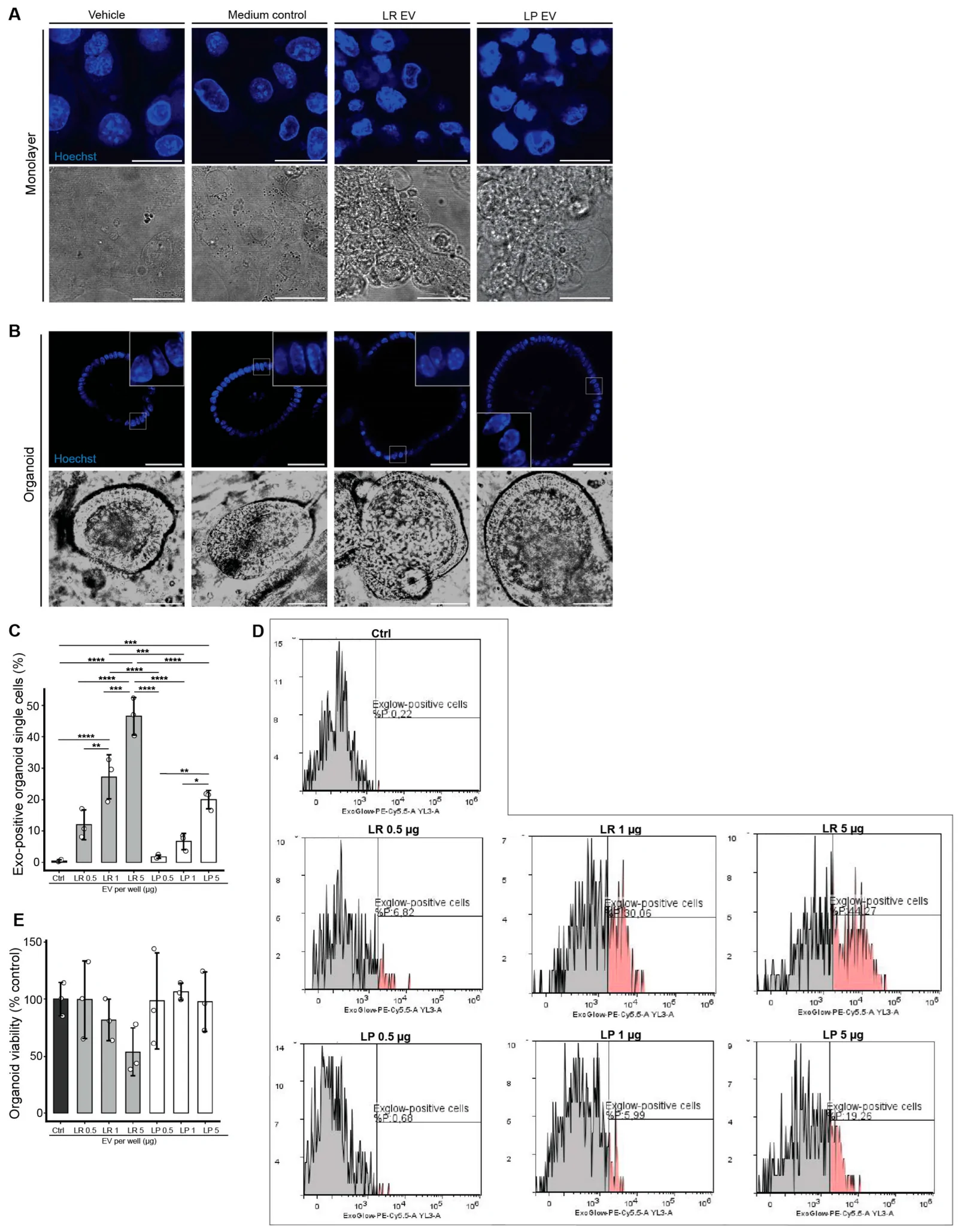
EV uptake is dose-dependent and inversely correlates with organoid viability. (**A**,**B**). Nuclear (**top row**) and brightfield imaging (**bottom row**) of colonic monolayers ((**A**), scale bar 25 µm) and organoids ((**B**), bottom row, scale bar 50 µm) incubated with DPBS (“Vehicle”), non-inoculated concentrated MRS medium (“Medium control”), 5 µg LR or LP EVs, respectively, for 6 h at 37 °C. Nuclei were counterstained with Hoechst. As opposed to vehicle and medium-only controls, colonic monolayers exposed to LR or LP EVs appear severely damaged, pointing to EV-induced cytotoxicity. (**C**) Quantitative flow cytometry analysis of EV internalization by colonic organoids previously incubated with 0.5, 1, or 5 µg LR- or LP-EV for 6 h at 37 °C and dissociated into single cells. DPBS served as a control (EV vehicle). EV uptake showed a dose-dependent increment for both species, with LR EVs consistently showing higher uptake rates than LP EVs across the entire dose range. (**D**) Representative histograms depicting the proportion of Exo^+^ colonic organoid single cells (highlighted in red). (**E**) Viability of organoids treated as described in (**A**) was quantified with a 3D ATP-based luminescence assay. Viability of LR-EV-treated organoids declined progressively with increasing LR EV doses. Results were reported as % of control condition. For panels (**C**,**E**), data represent *n* = 3 mice (*n* = 4 technical replicates). One-way ANOVA followed by Tukey’s multiple comparison test (*p*-value: ns > 0.05; * < 0.05; ** < 0.01; *** < 0.001; **** < 0.0001). Bars indicate mean ± SD.

Following incubation with LR- and LP-derived EVs, epithelial monolayers were partially detached, and cells had adopted a rounded shape, whereas monolayers treated with vehicle or concentrated bacterial culture medium remained macroscopically unaffected. Consistently, nuclei in LR- and LP EV-exposed monolayers displayed signs of segmentation, chromatin condensation and irregular contours, while control-treated monolayers retained a normal nuclear architecture (Figure 5A). By contrast, colonic organoids appeared intact regardless of the treatment condition (Figure 5B). To verify EV internalization by organoids, we subsequently performed flow cytometry to quantify uptake of ExoGlow (Exo)-labeled LR and LP EVs (Figure 5C,D). Exo is a lipophilic fluorophore originally developed for incorporation into human exosome membranes. This dye was chosen for its crisp signal and rapid labeling procedure, obviating the need for repeated ultracentrifugation to purify labeled EVs. In the absence of EVs, Exo induced a weak non-specific membrane signal likely arising from dye micelles interacting with the host cell membrane (Figure S5). Across a range of ascending EV amounts, we noticed a gradual increase in EV incorporation into epithelial cells, consistent with a linear dose–uptake relationship which could be demonstrated for both species. We observed that uptake rates of LR EVs were always significantly higher than those of LP EVs with any quantity of EV added to the organoids. In parallel, we monitored organoid viability, applying the same EV dosing scheme (Figure 5E). Following incubation with escalating amounts of LR EVs, a non-significant decline in ATP-synthesizing organoids was noted, while viability of LP EV-treated organoids remained unchanged throughout the range of tested EV doses.

**Figure 6:**
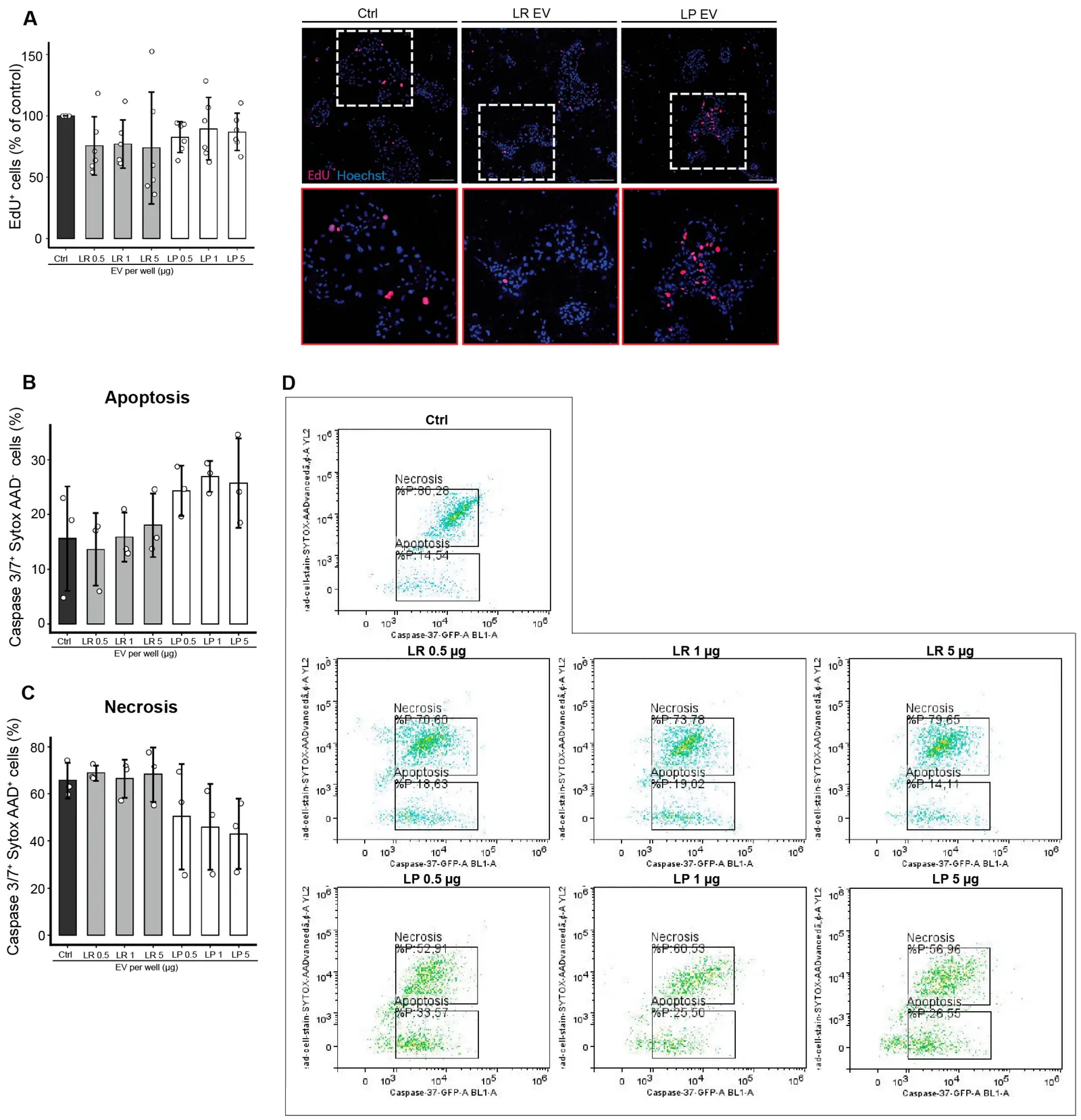
LP EVs skew cell fate of distressed organoids towards apoptosis. (**A**) DNA synthesis of colonic epithelial monolayers treated with 0.5, 1, or 5 µg LR- or LP-EV for 6 h at 37 °C was measured by EdU incorporation. For quantification, the count of EdU^+^ cells per 1000 cells was averaged from *n* = 10 random areas of colonic epithelial monolayers from *n* = 3 mice. EdU^+^ cells trended towards lower counts in the presence of 5 µg LR EVs compared to LP EVs (**left**). Interspersed EdU^+^ cells (red) were visualized within the colonic epithelial monolayer (scale bar 50 µm) (**right**). (**B**–**D**) Fate of colonic organoid cells treated as described in (**A**) was evaluated following infliction of epithelial damage by mechanical dissociation. Single cells were analyzed via flow cytometry and gated for Caspase 3/7^+^, Sytox AAD^−^ (apoptosis) and Caspase 3/7^+^, Sytox AAD^+^ (necrosis) populations. Results were reported as % of the control condition. In the presence of LP EVs, mechanically disrupted colonic organoid cells were more prone to apoptosis irrespective of the EV amount (**B**), while a compensatory decrease in necrotic cells was noted (**C**). Representative histograms showing single organoid cells gated for apoptosis and necrosis, respectively (**D**). For panels B and C, data represent *n* = 3 mice (*n* = 4 technical replicates). One-way ANOVA followed by Tukey’s multiple comparison test (*p*-value: ns > 0.05). Bars indicate mean ± SD.

To further investigate this decline in cellular viability, we next asked whether bacterial EVs might impact the EdU^+^ proliferative niche of the colonic epithelium (Figure 6A). Actively dividing cells, predominantly intestinal stem cells, tended to enter S phase less frequently in response to LR EVs, particularly when exposed to higher EV amounts. Data interpretation was hampered by a high variability in the number of EdU^+^ cells per field of view, which may be explained by epithelial cells growing in stacks, impeding even penetration by Exo-labeled EVs.

Furthermore, we investigated whether EVs modulate cell fate decisions in colonic epithelium subjected to acute injury through mechanical disintegration (Figure 6B–D). Cell death was found to be shifted in favor of apoptosis in LP EV-treated organoids without resulting in a statistically significant difference. This predisposition for undergoing apoptosis seemed to be independent of the administered EV dose. By contrast, the fraction of apoptotic cells was not altered in the presence of LR EVs.

In summary, we observed that LR-derived EVs, even if administered in low amounts, are internalized by colonic epithelial cells more efficiently than LP-derived EVs. Elevated uptake rates of LR EVs were found to coincide with a lower level of organoid viability. Due to a high variability in normalized EdU^+^ counts, we were unable to provide robust evidence that this finding is associated with a reduction in epithelial DNA replication activity. In addition, EVs interfere with cellular homeostasis by skewing host cell fate in a species-dependent manner towards apoptosis in LP EV-treated organoids.

### 3.5. The Proteomic Profile of LR EV-Treated Colonic Organoids Resembles the Cytopathological Signature of Neurodegenerative Disorders

**Figure 7:**
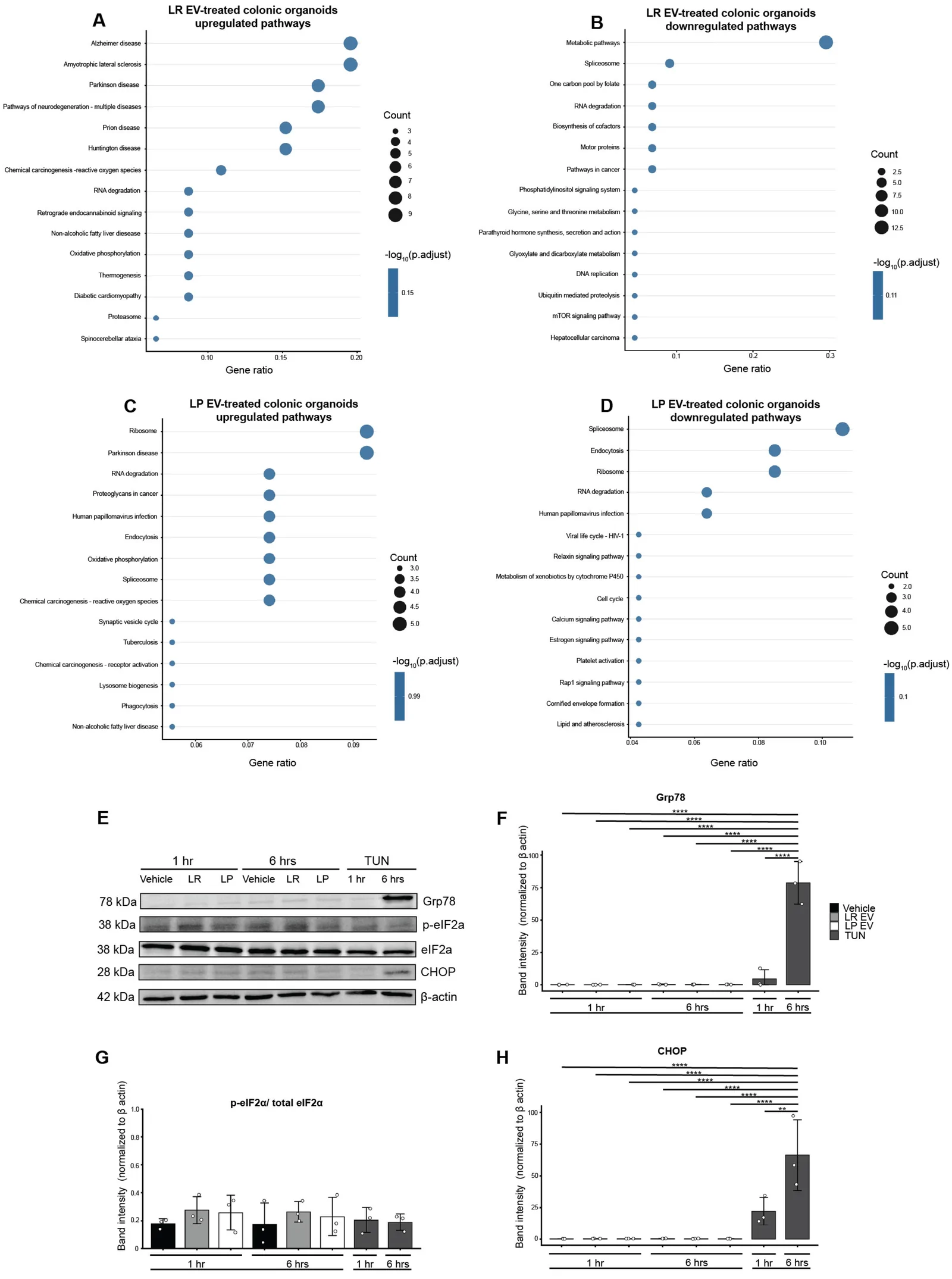
The proteomic profile of LR EV-treated colonic organoids resembles the cytopathological signature shared by neurodegenerative disorders. (**A**–**D**). Differential regulation of eukaryotic KEGG pathways in colonic organoids treated with 5 µg LR EV or LP EV for 6 h (*n* = 3 mice, *n* = 2 technical replicates). Proteins with |log2 fold change| ≥ 1 and *p* < 0.05 were selected for downstream over-representation analyses. The x-axis of the dot plot displays fold enrichment. Dot size and coloring denote the gene count and the Benjamini–Hochberg-adjusted −log10(*p*-value) level, respectively. Upregulated KEGG pathways in LR EV-treated organoids (**A**), downregulated KEGG pathways in LR EV-treated organoids (**B**), upregulated KEGG pathways in LP EV-treated organoids (**C**), and downregulated KEGG pathways in LP EV-treated organoids (**D**). (**E**–**H**). Western blot probing unfolded protein response (UPR)-associated regulatory proteins (BiP/Grp78, CHOP, phospho-eIF2α, and total eIF2α) in colonic organoids treated with DPBS (vehicle), 5 µg LR or LP EVs for 1 and 6 h, respectively (*n* = 3 mice). Organoids exposed to tunicamycin (TUN, 0.5 µg/mL) for the same time periods served as positive controls for UPR. Band intensities were normalized to β-actin (**E**). Densitometry analysis of BiP/Grp78 (**F**), ratio of phospho-eIF2α/total eIF2α (**G**), and CHOP (**H**). One-way ANOVA followed by Tukey’s multiple comparison test (**F**–**H**). Statistical significance was determined using Benjamini–Hochberg-adjusted *p*-values (ns > 0.05; ** < 0.01; **** < 0.0001). Bars indicate mean ± SD.

Next, we performed proteomic analysis of colonic organoids challenged with LR or LP EVs to explore their impact on intracellular signaling cascades. The list of identified proteins and protein abundances is included in Table S5. Results from differential abundance analysis are shown in Figure S6A,B and Table S6, respectively. Functional annotation of differentially regulated proteins by GO enrichment revealed that LR EV-treated organoids were enriched in cellular function terms involving “Cytoplasmic stress granules” and “Proteasome complex”. Conversely, biological functions essential for cellular RNA splicing, including “Catalytic step 2 spliceosome”, “Ribonucleoprotein complex”, and “Spliceosomal complex,” were downregulated. Colonic organoids exposed to LP EVs exhibited enrichment for laminin trimers, while the remainder of functional terms did not show remarkable regulation patterns (Figure S6C,D, Table S7). Subsequent KEGG pathway analysis revealed that LR EV-treated colonic organoids exhibited enrichment in the terms “Alzheimer disease”, “Amyotrophic lateral sclerosis”, “Parkinson disease”, “Prion disease”, “Huntington disease,” as well as the overarching term “Pathways of neurodegeneration—multiple diseases,” while the term “Metabolic pathways,” comprising multiple core biochemical processes, was decreased. Besides an isolated upregulation of the term “Parkinson disease”, LP-treated colonic organoids again did not exhibit a distinct proteomic signature (Figure 7A–D, Table S8). Because the term “Pathways of neurodegeneration—multiple diseases” encompasses disorders which are, among others, characterized by pathological protein accumulation, we hypothesized that exuberant influx of bacterial proteins trafficked within EVs might elicit a similar proteomic response. To further explore the findings obtained from mass spectrometry, we performed Western blot to evaluate whether EV exposure could trigger activation of the unfolded protein response (UPR). Following incubation with either LR- or LP-derived EVs, no early increase in the phosphorylated eIF2α/total eIF2α ratio was observed, and expression levels of BiP/Grp78 and CHOP remained persistently low at 6 h, indicating that UPR activation is unlikely to account for the observed impairment of organoid metabolism (Figure 7E–H).

Collectively, GO term and KEGG pathway enrichment analyses suggest that, in particular, LR EV exposure induces an enrichment of proteomic pathways associated with cellular stress and pathways annotated to neurodegenerative disorders.

### 3.6. EVs Enter Colonic Epithelium upon Addition of Strain-Matched LTA to a Lesser Extent and Are Partially Transported to Acidic Endo-Lysosomal Compartments

**Figure 8:**
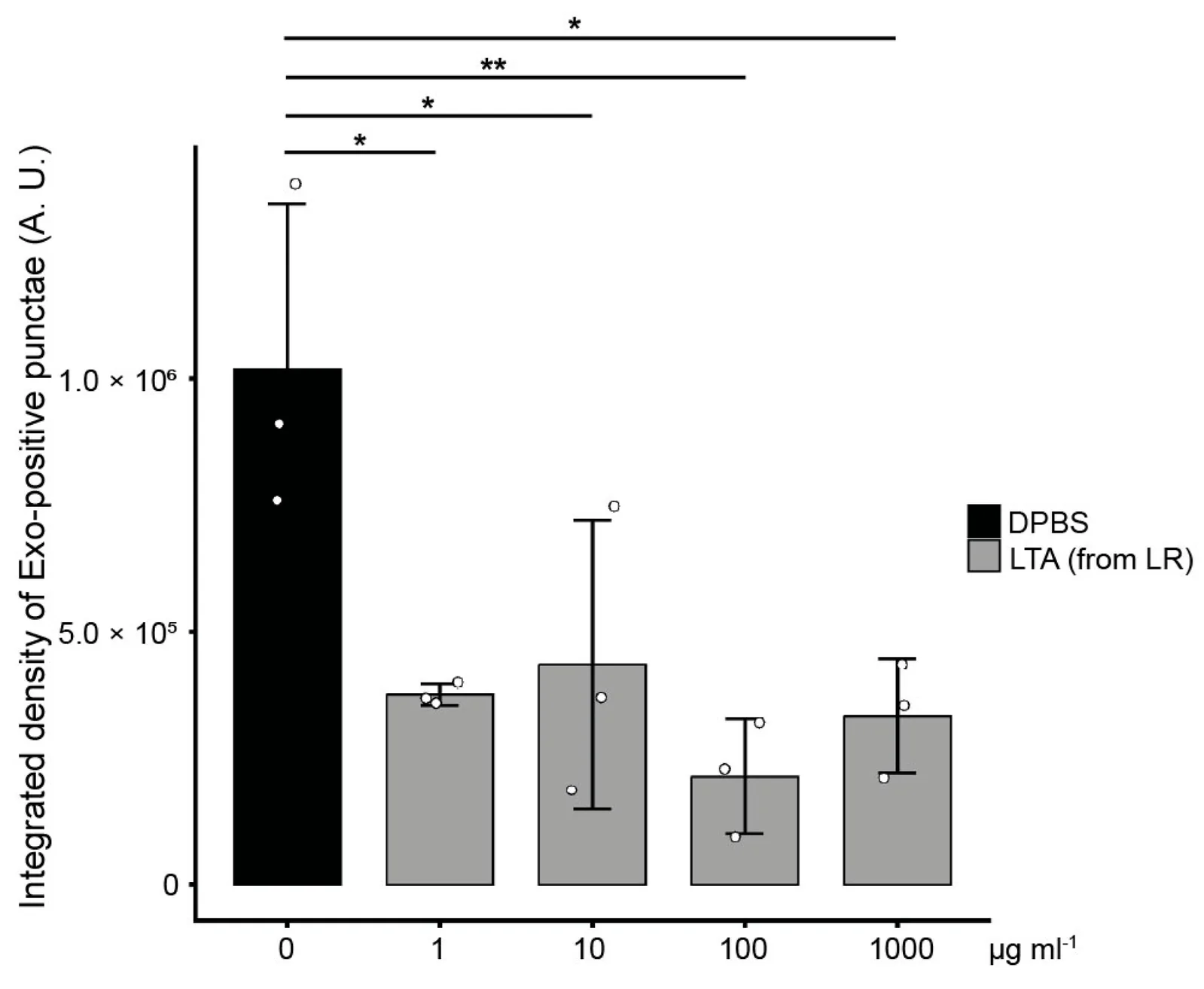
Previous saturation of colonic epithelium with LTA counteracts internalization of EVs. Colonic monolayers were pretreated with solubilized LTA isolated from the parental LR strain (DPBS as vehicle, 1 µg/mL, 10 µg/mL, 100 µg/mL, 1000 µg/mL) for 30 min before Exo-LR EVs were added at 0.05 µg per well for another 60 min. For quantification, EV uptake was assessed in *n* = 3 mice (*n* = 10 individual cells per mouse). Integrated fluorescence density of Exo-positive punctae was plotted on the y-axis. One-way ANOVA followed by Tukey’s multiple comparison test (*p*-value: ns > 0.05; * < 0.05; ** < 0.01). Bars indicate mean ± SD.

Given the presence of vesicle-bound LTA, we hypothesized that LTA might act as a ligand facilitating EV docking to the host cell membrane. EVs were masked with an LTA-specific monoclonal antibody applied at varying concentrations. EVs from LR, as the representative species, were used to perform competitive blocking of EV binding to the host cell membrane. Given the presence of LTA on EVs from either species, the underlying mechanism of action was assumed to equally apply to LP-derived EVs. Irrespective of the concentration, anti-LTA pretreatment significantly reduced the internalization of LR EVs into the colonic epithelium. However, treatment of monolayers with a class-matched isotype antibody elicited the same effect, pointing to non-specific antibody-mediated agglutination of EVs (Figure S7). Therefore, we performed saturation of the epithelial cell membrane with LTA isolated from the identical parental strain, resulting in decreased EV uptake across all tested LTA concentrations (Figure 8 and Figure S8). The latter finding may provide supportive evidence that LTA moieties contained within the vesicle membrane may be involved in EV entry into the colonic epithelium.

**Figure 9:**
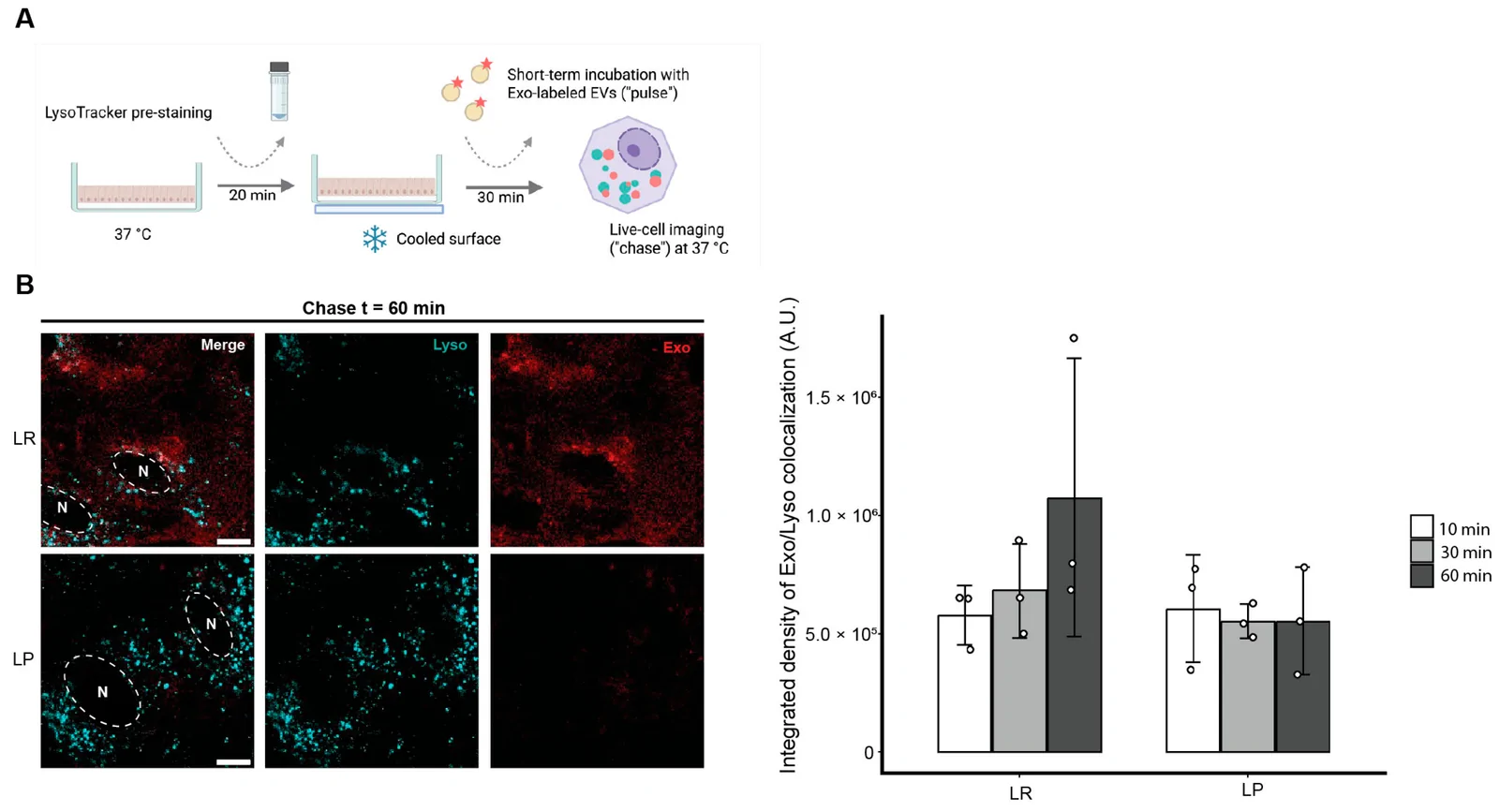
EVs adhere to the epithelial membrane and are partially trafficked to acidic endo-lysosomal compartments. (**A**,**B**). To visualize intracellular trafficking, colonic epithelial monolayers were first pre-stained with Lysotracker Blue (LT) at 37 °C and then exposed to a pulse of 5 µg Exo-labeled EVs for 30 min while being maintained on ice. Following replacement with warmed EV-free medium, live-cell confocal imaging was performed under standard culture conditions (37 °C, 5% CO_2_) to monitor EV internalization. A scheme of the experimental setup was created with Biorender.com (**A**). *Left*: Endo-lysosomal structures appear as fine speckles centered around the nucleus, which in parts co-localize with the fluorescence signal emitted by Exo-labeled EVs (representative images acquired after 60 min of live-cell imaging). Nuclei (N) correspond to vacant intracellular zones (scale bar 10 µm). *Right*: For quantification, *n* = 3 mice (*n* = 10 individual cells per mouse) were iteratively analyzed. Integrated fluorescence density of the Exo/LT colocalization signal per cell was plotted on the y-axis (**B**). Confocal images shown in (B) were acquired with a Leica SP8 system. One-way ANOVA followed by Tukey’s multiple comparison test (*p*-value: ns > 0.05). Bars indicate mean ± SD.

Lastly, to track the fate of EVs inside the host cell, acidified endo-lysosomal compartments were flagged with a pH-sensitive dye before Exo-labeled EVs were added (Figure 9A). Colonic epithelial monolayers were temporarily cooled down and incubated with Exo-labeled LR or LP EVs (“pulse”) to allow attachment of EVs to the cell membrane without being internalized. Synchronized internalization was initiated upon re-warming. At 10 and 30 min after resuming cultivation at 37 °C (“chase”), no difference in the co-localization signal was noted regardless of the species. After 60 min, a higher but not significant co-localization signal was observed in LR EV-treated epithelial cells (Figure 9B). These results demonstrate that once internalized, a small fraction of EVs is sorted into acidic endo-lysosomal compartments, and support the previous observation of higher uptake efficiency of LR EVs versus LP EVs.

## 4. Discussion

In recent years, bacterial EVs have garnered increasing attention as key mediators of inter-kingdom communication, functioning as messenger particles between microbes and the host. While early studies highlighted the potent immunomodulatory capacity of EVs derived from both commensal and pathogenic gut bacteria—particularly in their interactions with various immune cell subsets—the role of the intestinal epithelium, as the initial barrier encountered by luminal EVs, has remained comparatively underexplored. A limited number of studies have demonstrated that bacterial EVs are capable of crossing the epithelial barrier [44] and accumulating within epithelial cells [13,14]. However, our understanding of how bacterial EVs specifically affect primary colonic epithelial cells, and whether these effects differ across bacterial taxa and environmental contexts, remains elusive.

To bridge this gap, the present study was designed to concurrently examine two probiotic LAB species, LR and LP, which belong to separate genera within the *Lactobacillaceae* family. This approach ensured adequate phylogenetic distance while still preserving taxonomic concordance at the family level. Physicochemical characterization of EVs derived from LR and LP revealed particle size distributions consistent with prior studies profiling EVs from *Lactobacillus acidophilus* ATCC 53544, *Lactobacillus casei* ATCC 393, and *Lactobacillus reuteri* ATCC 23272 under standardized culture conditions [38]. Similarly, zeta-potential measurements indicated a net negative surface charge with notable interspecies variability.

In a subsequent step, we directed attention to long-chain fatty acids and LTA type 1 as principal structural components of the bacterial cytoplasmic membrane and, by extension, of the EV membranes. Membrane fluidity of bacterial membranes is known to be shaped both by species-inherent differences [45] and responsiveness towards environmental factors through dynamic modulation of the fatty acid composition [46,47]. Notably, such adaptive processes can alter the saturated-to-unsaturated fatty acid ratio and newly introduce trans-unsaturated fatty acids, as observed in EV membranes from *Komagataeibacter oboediens* when cultivated under extraterrestrial conditions [48]. In our study, mass spectrometric analysis of EV membrane lipids showed that both LR and LP EVs contain the saturated LCFA stearic acid (C18:0) and, in traces, arachidic acid (C20:0), tridecanoic acid (C13:0), and the monounsaturated LCFA *cis*-11-eicosenoic acid (C20:1 (11Z)), while the remainder of LCFAs is detectable in both inoculated and non-inoculated medium preparations. We next investigated whether the EVs from LR and LP differ in the degree of LTA incorporated in their membranes. Earlier studies had reported the presence of LTA in EVs from *Lacticaseibacillus rhamnosus* JB-1 MV [13] and *Lactobacillus gasseri* JCM 1131T [14], but not in those from *Lactiplantibacillus plantarum* JCM8341 [16]. In our study, LTA was detected in EVs from both LR and LP, without, however, resulting in a species-dependent difference in quantity. However, it cannot be ruled out that the LTA molecules of LR and LP bacteria, including the backbone and substituents, differ in a way sufficient to significantly influence the zeta potential, as has previously been demonstrated for *dltA* mutant *Staphylococcus* species [49]. We further characterized the proteomic cargo of EVs, particularly focusing on proteins predicted to localize either to the cytoplasmic membrane or be secreted into the extracellular space, as these may serve as potential anchoring elements to the host cell membrane. In several studies, Lpps have been reported to play a crucial role in bacterial adhesion to and invasion of intestinal epithelial cell lines [50,51,52]. We could ascertain the presence of Lpps in EVs from either species, which qualify as candidate proteins ligating EVs to the host colonic epithelium.

We confirmed that EVs from both LR and LP are internalized not only by transformed intestinal epithelial cell lines, such as Caco-2 cells as previously reported [6,8,38,53], but also by primary colonic epithelial cells. Our results revealed a marked difference in uptake efficiency between the two species, which persisted across a range of EV amounts. To our knowledge, this is the first study to report such a disparity, which may extend beyond the specific strains under investigation. Systematic inter- and intra-species comparative studies involving multiple strains are needed before results can be generalized. We noted an inverse correlation between the uptake efficiency of bacterial EVs and viability of the colonic epithelium. Concurrently, a trend towards declining numbers of DNA-replicating cells treated with LR EVs was observed. Preceding studies demonstrated DNA damage-induced cytotoxicity inflicted by incubation with EVs not only from bacterial pathogens such as pathogenic adherent-invasive and enterohemolytic *Escherichia coli* but also from the avirulent strain Seattle 1946 [6,53]. In our study, proteome-based enrichment analysis of LR EV-treated colonic organoids showed a decrease in the functional super-term “Metabolic pathways” whilst GO terms including “Cytoplasmic stress granule” and “Proteasome complex” were overrepresented. Cytoplasmic stress granules have been ascribed a beneficial role by sheltering mRNA and other cargo, including kinases and phosphatases during stress events [54]. More recent evidence specifically reported enhanced toxicity in several *Drosophila* models of neurodegenerative disorders when stress granule formation was experimentally inhibited [55]. Furthermore, inspection of differentially regulated KEGG pathways revealed a particular enrichment of neurodegenerative disorders, intertwining different cellular pathologies such as endoplasmic reticulum stress and UPR, formation of free radicals/reactive oxygen species, mitochondrial dysfunction, and autophagy, in LR EV-treated colonic organoids. Whilst our results argue against a contribution of UPR to the observed EV-induced alterations, oxidative distress and mitochondrial dysfunction may be more plausible underlying mechanisms, consistent with a slight enrichment in the KEGG term “Chemical carcinogenesis—reactive oxygen species” in EV-treated colonic organoids as well as prior reports [56,57]. Importantly, these pathway enrichment analysis results represent exploratory findings requiring more in-depth experimental validation. The increased susceptibility to apoptosis versus necrosis in dissociated colonic organoids treated with LP EVs may reflect a higher metabolic competence and preserved ATP availability in these cells compared with LR EV treatment [58,59].

Using LR strain-matched LTA, LR EV entry into colonic epithelium was decreased to some degree, suggesting that LTA inserted in the EV membrane may promote adhesion to the host colonic epithelium. Granato et al. reported that LTA expressed on the surface of *Lactobacillus johnsonii* La1 was identified as a crucial adhesion factor for reversible attachment of bacterial cells to the colonocyte-like Caco-2 cell line [60]. Similar results were reported from another study comparing adhesion of *Lactobacillus plantarum* A3, *Lactobacillus reuteri* DMSZ 8533, and *Lactobacillus acidophilus* CICC 6074 to Caco-2 cells, revealing significant species-dependent differences [61]. In fact, LTA could act synergistically with surface Lpps in a two-tier process, loosely tacking bacterial EVs to the host cell membrane before being replaced by a more specific protein receptor interaction [62,63]. By contrast, attempts to prevent EV binding to the epithelial membrane by masking with anti-LTA antibodies remained inconclusive, which might be related to the presence of a mucus-binding protein with IgG immunoglobulin-binding properties, as previously described in *Lactobacillus reuteri* strain ATCC 1063 [64]. Again, aligning with earlier studies [65,66], our findings showed that following internalization, LR and LP EVs are partially trafficked to acidic endo-lysosomal structures.

This study has some limitations. In general, the experimental design follows a descriptive approach and the intention to address several sequential events of the interaction process between bacterial EVs and colonic epithelium, including binding, entry, intracellular trafficking, and impact on cellular biology, has entailed an overall lack of mechanistic depth. For example, the study is not suited to derive mechanistic proof, in particular regarding the role of LTA as a ligand for Gram-positive bacteria-derived EVs to attach to the epithelial membrane. Preferably, studies with an LTA mutant strain would be required to compare internalization efficiency with the corresponding wild-type strain, flanked by zeta-potential measurements and structural analysis of the LTA molecule.

The interaction between bacterial EVs of two *Lactobacillus* species and colonic epithelium was studied in a highly simplified environment in the absence of resident microbiota, an epithelial mucus layer, stromal, and immune cells, all of which modulate the intestinal mucosal barrier. While microbial EVs are commonly quantified as particles per g feces or particles per mL luminal fluid, the total amount of luminal bacterial EVs indexed to the intestinal surface can only be approximated [67,68]. The use of EVs from *Lactobacillus* species originally isolated from the murine colon would further increase the physiological relevance of the model. To overcome the limitation of varying biological responses of organoids from individual animals, a higher number of biological replicates should be chosen a priori. Lastly, the translational potential of the results could be strengthened by using human colonic organoids, preferably integrated into a more complex co-culture system.

## 5. Conclusions

In this study, we sought to characterize the properties of EVs derived from the LAB species LR and LP. Adopting a comparative approach, we aimed to investigate how EVs from distinct Gram-positive bacterial species interact with the colonic epithelium, which, unlike in earlier studies, is derived from mammalian organoids to better recapitulate cell physiology. We found that EVs derived from different bacterial species differ in their ability to enter the colonic epithelium. The presumed role of EV-bound LTA to mediate internalization could not be definitively elucidated. Also, we demonstrate at the proteomic level that exuberant internalization of LR EVs is accompanied by overrepresentation of a cellular pathway signature shared by neurodegenerative diseases. Insights gained from this study will hopefully aid in identifying factors modulating bacterial EV internalization and could be used to repurpose the pre-existing envelope of non-pathogenic EVs to deliver therapeutics into host cells.

## Data Availability

The original mass spectrometry proteomics data presented in the study are openly available in ProteomeXchange Consortium via the PRIDE partner repository (accession number PXD067937).

## Supplementary Materials

The following supporting information can be downloaded at: https://www.mdpi.com/article/10.3390/microorganisms14102367/s1. Figure S1: LR and LP bacteria exhibit a similar growth rate under identical culture conditions. (A) Growth curves of LR (solid line) and LP (dashed line) bacteria cultivated at 37 °C, 21% O_2_ in De Man-Rogosa-Sharpe (MRS) medium (*n* = 6 independent replicates per species). The x-axis depicts optical density (OD) 600 while the y-axis shows the elapsed time of cultivation (in hours). Prior to OD600 measurements, bacterial suspension samples were diluted 1:8 in non-inoculated MRS medium to maintain readings within the linear range. The red square highlights the target OD600 range of 4.0–5.0. Figure S2: EV preparations are sterile with no relevant particle contamination from the MRS medium. (A) Line plots of representative Nanoparticle Tracking Analysis (NTA) readings obtained from non-inoculated MRS medium following SF (gray) and UC (black), LR-inoculated MRS medium following SF (light red) and UC (dark red), and LP-inoculated MRS medium following SF (light blue) and UC (dark blue). Lines indicate mean values (*n* = 3 technical replicates); gray shaded area denotes SD. (B) Particle-to-protein ratio of LR and LP EV preparations (*n* = 3 independent preparations). (C) MRS agar plates inoculated with LR EVs and LP EVs for 24 h at 37 °C showed no growth of bacterial colonies. Plates inoculated with DPBS, non-inoculated MRS medium and viable LR and LP bacteria served as negative and positive controls, respectively. Figure S3: LR and LP EVs significantly differ in their zeta potential. (A) Line plots of *n* = 3 representative zeta-potential readings of EVs produced by LR and LP, measured by electrophoretic light scattering (LR, dashed line; LP, solid line). The x-axis denotes the zeta-potential (in V), while the y-axis depicts the relative frequency of a given zeta-potential value. Electrophoretic mobility (y-axis scaled in μm × cm/V × s) (B) and conductivity (y-axis scaled in mS/cm) (C) of LR and LP EVs. All measurements were obtained from *n* = 7 independent EV preparations. Statistical comparisons were performed using Welch’s corrected *t*-test (*p*-values: ns > 0.05; ** < 0.01). Bars indicate mean ± SD. Figure S4: The proteome of LR and LP EVs and parental bacteria mainly differs by type of sample material rather than species. (A) Representative lysates of EVs and bacterial cells from LR and LP were separated by SDS-PAGE and stained with Coomassie brilliant blue dye. Resulting protein band patterns were similar between LR and LP EVs but markedly differed between bacterial cells of either species. (B) Venn diagrams display the overlap between proteins isolated from LR and LP bacterial cells and EVs. (C) Principal component analysis of LR and LP bacterial cells and EVs (*n* = 3 independent replicates per species and sample type) was calculated from log2-transformed (log2[x + 1]) protein intensity values and revealed sample type as the major source of separation (PC1: 71.3% explained variance). (D) Sample-to-sample distance heatmap illustrating pairwise Euclidean distance-based hierarchical clustering of log2-transformed protein intensity values. Color intensity (blue: low; red: high) encodes the magnitude of dissimilarity in protein composition between two samples. Figure S5: Host cell membranes are non-specifically stained by EVs labeled with the lipophilic dye Exo. Colonic organoids and monolayer cultures were incubated with non-inoculated medium (medium control), Exo-DBPS (dye-only), 5 µg Exo-LR EVs, and 5 µg Exo-LP EVs for 6 h at 37 °C. Control samples were processed identically to the EV preparations and were added in equivalent volumes. Exo channel (white, *top row*) and Hoechst channel (blue, *bottom row*). Scale bars are 50 µm. Figure S6. Colonic organoids treated with LR EVs are enriched in functional terms related to stress granule formation and the proteasome. (A,B) Volcano plot showing the log 2-fold change (x-axis) against −log 10 *p*-value (y-axis) for all differentially expressed proteins (DEP) between LR EV-treated organoids versus control (A) and between LP EV-treated organoids versus control (B). A |log 2-fold change| ≥ 1 and *p* < 0.05 were considered statistically significant. All significantly regulated DEPs are highlighted in red (upregulated) or blue (downregulated). (C,D) Significantly regulated DEPs detected in LR EV- (C) or LP EV- (D) treated colonic organoids were consecutively analyzed by Gene Ontology (GO) using the web-based DAVID bioinformatics resource for functional annotation. Results were categorized by the top Gene Ontology (GO) terms “Biological Process” (BP), “Molecular Function” (MF), and “Cellular Component” (CC). Analysis was performed on colonic organoid samples from *n* = 3 mice (*n* = 2 technical replicates). The x-axis displays fold enrichment using a symmetric logarithmic transformation [sign(x) × log10(1 + |x|)], while axis labels indicate the original fold-enrichment values. Dot size and coloring denote the number of genes and the Benjamini–Hochberg-adjusted *p*-value level, respectively. For visualization, the 10 GO terms with the lowest Benjamini-corrected *p*-values were selected separately for each GO category and direction of regulation, with fold enrichment used as a secondary ranking criterion. Figure S7: Pre-incubation of LR EVs with anti-LTA antibody or isotype interferes with their internalization by colonic epithelium. (A) Exo-LR EVs were masked by overnight incubation with either monoclonal anti-LTA antibody or class-matched isotype (DPBS as vehicle, 10 μg/mL, 1 μg/mL, 0.1 μg/mL, 0.01 μg/mL) before being added at 0.05 μg to colonic epithelial monolayers for 60 min. For quantification, EV uptake was assessed in *n* = 3 mice (*n* = 10 individual cells per mouse). Integrated fluorescence density of Exo-positive punctae was plotted on the y-axis. One-way ANOVA followed by Tukey’s multiple comparison test. Statistical significance was determined using Benjamini–Hochberg-adjusted *p*-values (ns > 0.05; * < 0.05; ** < 0.01). Bars indicate mean ± SD. Figure S8: ^1^H NMR-based structural resolution of LR-derived LTA. ^1^H NMR spectrum of crude LT from LR was recorded at 298 K using D_2_O as a solvent, with the residual solvent peak at 4.79 ppm as reference. Residual impurities were tolerated, as fine structural resolution of substituent groups and calculation of signal integrals were not intended. Unsubstituted glycerol groups (-CH2, -CH) are expected in the range δH 3.94–4.12 ppm according to (1), supporting the presence of the LTA glycerophosphate backbone (region highlighted in orange). D_2_O, heavy water. Table S1: Vesicle membranes from LR and LP EVs share a similar composition of long-chain fatty acids. Long-chain fatty acids (LCFA) detected by gas chromatography/mass spectrometry (GC-MS) in the lipid phase from LR and LP EV preparations (Medium control: non-inoculated concentrated MRS medium) are listed in descending order (*n* = 3 independent replicates). Concentrations (mean ± SD) are reported in µg/mL. Table S2: Top ten most abundant proteins of LR and LP EVs and parental bacterial cells predicted to be localized in the cytoplasmic membrane. For categories comprising fewer than ten proteins, only the most abundant representatives were reported. Proteins are ranked based on the cumulative abundance across *n* = 3 independent replicates. LP, *Lactiplantibacillus plantarum* (strain WCFS1); LR, *Limosilactobacillus reuteri* (strain DSM 20016). Table S3: Top ten most abundant proteins of LR and LP EVs and parental bacterial cells predicted to be excreted into the extracellular environment. For categories comprising fewer than ten proteins, only the most abundant representatives were reported. Proteins are ranked based on the cumulative abundance across *n* = 3 independent replicates. LP, *Lactiplantibacillus plantarum* (strain WCFS1); LR, *Limosilactobacillus reuteri* (strain DSM 20016). Table S4: Summary of identified proteins and protein abundances in bacterial EVs and pellets. Data compiled from Proteome Discoverer software matching against the UniProt reference databases of *Limosilactobacillus reuteri* strain DSM 20016 (UP000001991) and *Lactiplantibacillus plantarum* strain WCFS1 (UP000000432). Table S5: Summary of identified proteins and protein abundances in EV-treated colonic organoids. Data compiled from Proteome Discoverer software matching against the UniProt reference database of Mus musculus (UP000000589). Table S6: Differential abundance analysis of EV-treated colonic organoid proteome data. Processed quantitative datasets include protein-wise log 2 fold changes, nominal *p*-values, and Benjamini–Hochberg-adjusted *p*-values. Technical replicates (*n* = 2) were previously averaged within biological replicates (*n* = 3 mice). Table S7: GO enrichment analysis of EV-treated colonic organoid proteome data. DAVID web tool-generated functional annotation chart displaying individual GO terms (Biological Process, Cellular Component, Molecular Function), gene counts, nominal and adjusted *p*-values. Table S8: KEGG pathway enrichment analysis of EV-treated colonic organoid proteome data. Processed quantitative datasets from overrepresentation analysis include gene ratio, fold enrichment, nominal and adjusted *p*-values.
