# Supplementary material for: A Descriptive Primary Cell-Based In Vitro System to Model Interactions Between Lactobacillus Extracellular Vesicles and Colonic Epithelium
Source: Microorganisms. 2026 Oct 15;14(10):2367. doi: 10.3390/microorganisms14102367 (PMC13707729; doi:10.3390/microorganisms14102367)
Supplement: Supplementary file 1 [file microorganisms-14-02367-s001.zip › Table S1.pdf]

| | | Concentration | | |
|---|---|---|---|---|
| **Informal name** | **Systematic name** | **LR** | **LP** | **Medium control** |
| Stearic acid | C18:0 | 7.10 ± 2.20 | 4.87 ± 0.68 | 0 |
| Methyl palmitoleinate | C17:1(9Z) | 3.14 ± 1.39 | 2.19 ± 0.56 | 0.56 ± 0.05 |
| Myristic acid | C14:0 | 2.24 ± 0.82 | 1.61 ± 0.39 | 1.64 ± 0.10 |
| *Cis*-11-eicosenoic acid | C20:1(11Z) | 0.48 ± 0.10 | 0 | 0 |
| *Cis*-10-heptadecenoic acid | C17:1(10Z) | 0.40 ± 0.21 | 0.23 ± 0.20 | 0.59 ± 0.02 |
| Heptadecanoic acid | C17:0 | 0.26 ± 0.06 | 0.11 ± 0.10 | 0.39 ± 0.03 |
| Dodecanoic acid | C12:0 | 0.26 ± 0.06 | 0.30 ± 0.15 | 0.89 ± 0.03 |
| Pentadecanoic acid | C15:0 | 0.22 ± 0.04 | 0.10 ± 0.08 | 0.16 ± 0.01 |
| Arachidic acid | C20:0 | 0.11 ± 0.20 | 0.09 ± 0.16 | 0 |
| Decanoic acid | C10:0 | 0.12 ± 0.01 | 0.11 ± 0.03 | 0.39 ± 0.02 |
| Undecanoic acid | C11:0 | 0.02 ± 0.02 | 0.02 ± 0,02 | 0.08 ± 0,01 |
| Tridecanoic acid | C13:0 | 0.03 ± 0.03 | 0 | 0 |

**Table S1**
