# Supplementary material for: A Descriptive Primary Cell-Based In Vitro System to Model Interactions Between Lactobacillus Extracellular Vesicles and Colonic Epithelium
Source: Microorganisms. 2026 Oct 15;14(10):2367. doi: 10.3390/microorganisms14102367 (PMC13707729; doi:10.3390/microorganisms14102367)
Supplement: Supplementary file 1 [file microorganisms-14-02367-s001.zip › Table S2 .pdf]

Accession ID	Protein name	Cumulative abundance	Sample	Species
F9UP51	Transcription regulator, TetR family	1.63E+09	EV	LP
F9UQ86	Large ribosomal subunit assembly factor BipA (GTP-binding protein BipA)	3.42E+08	EV	LP
Q88UU3	ATP synthase subunit beta (ATP synthase F1 sector subunit beta)	2.33E+08	EV	LP
Q88ZC1	Hypothetical membrane protein PlnT, membrane-bound protease CAAX family	1.43E+08	EV	LP
F9UMI5	Transcriptional attenuator, cell envelope-related, LytR family	7.33E+07	EV	LP
F9UQ75	ComE operon protein 1, DNA receptor	7.32E+07	EV	LP
F9UT98	Serine-type D-Ala-D-Ala carboxypeptidase	4.97E+07	EV	LP
F9UQQ2	Lipoprotein	4.52E+07	EV	LP
F9URD4	Extracellular protein	3.50E+07	EV	LP
Q06115	Bile salt hydrolase/transferase	3.49E+07	EV	LP

Q88UU3	ATP synthase subunit beta (ATP synthase F1 sector subunit beta)	2.17E+09	Bacteria	LP
F9UQ86	Large ribosomal subunit assembly factor BipA (GTP-binding protein BipA)	5.03E+08	Bacteria	LP
Q88ZC1	Hypothetical membrane protein PlnT, membrane-bound protease CAAX family	3.30E+08	Bacteria	LP
F9USG5	Multidrug ABC transporter, ATP-binding and permease protein	3.24E+08	Bacteria	LP
Q88Z31	ATP-dependent zinc metalloprotease FtsH	2.06E+08	Bacteria	LP
F9UNY1	Quaternary amine transport ATP-binding protein	1.58E+08	Bacteria	LP
P37063	Pyruvate oxidase (POX)	1.33E+08	Bacteria	LP
Q06115	Bile salt hydrolase/transferase (BSH/T)	1.03E+08	Bacteria	LP
F9UTR7	NADH dehydrogenase	7.88E+07	Bacteria	LP
F9UP45	Enoyl-[acyl-carrier-protein] reductase [NADH]	7.76E+07	Bacteria	LP

A5VK08	Penicillin-binding protein, 1A family	1.64E+09	EV	LR
A5VJ78	Large ribosomal subunit assembly factor BipA (GTP-binding protein BipA)	1.06E+09	EV	LR
A5VIR1	ATP synthase subunit beta (ATP synthase F1 sector subunit beta)	5.75E+08	EV	LR
A5VHY0	Substrate-binding region of ABC-type glycine betaine transport system	3.50E+08	EV	LR
A5VI91	Amino acid ABC transporter ATP-binding protein, PAAT family	3.20E+08	EV	LR
A5VKM9	Putative host cell surface-exposed lipoprotein Ltp-like helix-turn-helix region domain-containing protein	2.92E+08	EV	LR
A5VIV3	FAD-dependent pyridine nucleotide-disulfide oxidoreductase	2.74E+08	EV	LR
A5VMD3	PTS system IIA component, Glc family	2.41E+08	EV	LR
A5VJ73	Transketolase domain protein	1.86E+08	EV	LR
A5VKN8	Signal recognition particle protein (Fifty-four homolog)	1.55E+08	EV	LR

A5VIR1	ATP synthase subunit beta	5.51E+09	Bacteria	LR
A5VIJ3	Phosphate acetyltransferase	3.37E+09	Bacteria	LR
A5VJ73	Transketolase domain protein	2.32E+09	Bacteria	LR
A5VHP9	Na^+ dependent nucleoside transporter domain protein	1,43E+09	Bacteria	LR
A5VK74	3-oxoacyl-[acyl-carrier-protein] synthase 2	1.28E+09	Bacteria	LR
A5VJG6	Amino acid ABC transporter ATP-binding protein, PAAT family	1.11E+09	Bacteria	LR
A5VK68	Enoyl-[acyl-carrier-protein] reductase [NADH]	9.98E+08	Bacteria	LR
A5VIV3	FAD-dependent pyridine nucleotide-disulfide oxidoreductase	9.06E+08	Bacteria	LR
A5VKP4	Phosphate acyltransferase	8.92E+08	Bacteria	LR
A5VHP8	Amino acid/polyamine/organocation transporter, APC superfamily	8.59E+08	Bacteria	LR

Table S2
