# Supplementary material for: A Descriptive Primary Cell-Based In Vitro System to Model Interactions Between Lactobacillus Extracellular Vesicles and Colonic Epithelium
Source: Microorganisms. 2026 Oct 15;14(10):2367. doi: 10.3390/microorganisms14102367 (PMC13707729; doi:10.3390/microorganisms14102367)
Supplement: Supplementary file 1 [file microorganisms-14-02367-s001.zip › Table S3 .pdf]

Accession ID	Protein name	Cumulative abundance	Sample	Species
F9USJ3	Extracellular protein	1.20E+08	EV	LP
F9UPA5	Transpeptidase-transglycosylase (Penicillin binding protein 1A)	9.89E+07	EV	LP
F9UUA0	Extracellular protein,gamma-D-glutamate-meso-diaminopimelate muropeptidase	1.00E+07	EV	LP
F9UMN8	Lipoprotein, FMN-binding protein	9.38E+06	EV	LP
F9UQA0	Extracellular protein, NlpC/P60 family,gamma-D-glutamate-meso-diaminopimelate muropeptidase	6.20E+06	EV	LP
F9UQ85	Extracellular protein, cell-wall anchored	3.16E+06	EV	LP
F9URQ9	Serine-rich adhesin for platelets	1.01E+06	EV	LP

F9UTZ5	Bacteriocin peptide PlnJ	7.55E+08	Bacteria	LP
F9UPA5	Transpeptidase-transglycosylase (Penicillin binding protein 1A)	2.06E+07	Bacteria	LP
F9UMN8	Lipoprotein, FMN-binding protein	1.69E+07	Bacteria	LP
F9UUA0	Extracellular protein,gamma-D-glutamate-meso-diaminopimelate muropeptidase	1.45E+07	Bacteria	LP
F9URQ9	Serine-rich adhesin for platelets	2.99E+06	Bacteria	LP
F9USJ3	Extracellular protein	1.69E+06	Bacteria	LP

A5VL92	Mannosyl-glycoprotein endo-beta-N-acetylglucosamidase	1.53E+10	EV	LR
A5VL89	NLP/P60 protein	1.00E+10	EV	LR
A5VL90	N-acetylmuramoyl-L-alanine amidase, family 2	7.60E+09	EV	LR
A5VML7	Peptidoglycan hydrolase	7.21E+09	EV	LR
A5VL74	NLP/P60 protein	8.74E+08	EV	LR
A5VL73	Dextransucrase	4.23E+08	EV	LR
A5VLU8	Lipoprotein	4.28E+07	EV	LR
A5VJU8	N-acetylmuramoyl-L-alanine amidase, family 2	4.25E+07	EV	LR
A5VJT7	Peptidase M23B	1.11E+07	EV	LR
A5VKI8	NLP/P60 protein	6.41E+06	EV	LR

A5VML7	Peptidoglycan hydrolase	8.61E+08	Bacteria	LR
A5VL92	Mannosyl-glycoprotein endo-beta-N-acetylglucosamidase	5.47E+08	Bacteria	LR
A5VL90	N-acetylmuramoyl-L-alanine amidase, family 2	3,03E+08	Bacteria	LR
A5VL89	NLP/P60 protein	2.09E+08	Bacteria	LR
A5VL73	Dextransucrase	1.51E+08	Bacteria	LR
A5VLU8	Lipoprotein	1,17E+08	Bacteria	LR
A5VM13	Xaa-Pro dipeptidyl-peptidase	3.68E+07	Bacteria	LR
A5VJU8	N-acetylmuramoyl-L-alanine amidase, family 2	3.65E+07	Bacteria	LR
A5VL47	Peptidoglycan-binding LysM	2.88E+07	Bacteria	LR
A5VIL0	Peptidoglycan-binding LysM	1.96E+07	Bacteria	LR

Table S3
